# Leveraging complementary multi-omics data integration methods for mechanistic insights in kidney diseases

**DOI:** 10.1172/jci.insight.186070

**Published:** 2025-03-10

**Authors:** Fadhl Alakwaa, Vivek Das, Arindam Majumdar, Viji Nair, Damian Fermin, Asim B. Dey, Timothy Slidel, Dermot F. Reilly, Eugene Myshkin, Kevin L. Duffin, Yu Chen, Markus Bitzer, Subramaniam Pennathur, Frank C. Brosius, Matthias Kretzler, Wenjun Ju, Anil Karihaloo, Sean Eddy

**Affiliations:** 1 Department of Internal Medicine, Division of Nephrology, University of Michigan, Ann Arbor, Michigan, USA.; 2 Novo Nordisk A/S, Måløv, Denmark.; 3 Eli Lilly & Co., Indianapolis, Indiana, USA.; 4 Biopharmaceuticals R&D, AstraZeneca, Cambridge, United Kingdom.; 5 Johnson & Johnson, New Brunswick, New Jersey, USA.; 6 University of Arizona, Tucson, Arizona, USA.; 7 Novo Nordisk Research Center Seattle, Inc, Seattle, Washington, USA.

**Keywords:** Nephrology, Chronic kidney disease, Expression profiling, Proteomics

## Abstract

Chronic kidney diseases (CKDs) are a global health concern, necessitating a comprehensive understanding of their complex pathophysiology. This study explores the use of 2 complementary multidimensional -omics data integration methods to elucidate mechanisms of CKD progression as a proof of concept. Baseline biosamples from 37 participants with CKD in the Clinical Phenotyping and Resource Biobank Core (C-PROBE) cohort with prospective longitudinal outcome data ascertained over 5 years were used to generate molecular profiles. Tissue transcriptomic, urine and plasma proteomic, and targeted urine metabolomic profiling were integrated using 2 orthogonal multi-omics data integration approaches, one unsupervised and the other supervised. Both integration methods identified 8 urinary proteins significantly associated with long-term outcomes, which were replicated in an adjusted survival model using 94 samples from an independent validation group in the same cohort. The 2 methods also identified 3 shared enriched pathways: the complement and coagulation cascades, cytokine–cytokine receptor interaction pathway, and the JAK/STAT signaling pathway. Use of different multiscalar data integration strategies on the same data enabled identification and prioritization of disease mechanisms associated with CKD progression. Approaches like this will be invaluable with the expansion of high-dimension data in kidney diseases.

## Abstract 2

In this proof-of-concept study, two complementary -omic integration approaches were used to identify complement and JAK-STAT pathways as shared signals and to prioritize mechanisms associated with kidney disease progression.

## Introduction

Chronic kidney diseases (CKDs) are a global public health concern and a risk factor for adverse outcomes in many diseases, including cardiovascular disease. In its 2022 report, the United States Renal Data System estimates that 14% of US adults have CKD and 2 in every 1,000 living persons are at risk of kidney failure (1). To address the large medical need in CKD, renin-angiotensin-aldosterone system blockers were developed and approved as first-line treatments in diabetic kidney disease (DKD) and CKD more than 2 decades ago (2–4). Recently, SGLT2 inhibitors and nonsteroidal mineralocorticoid receptor antagonists have been added to the armamentarium. These 2 new options have been specifically approved for diabetic and nondiabetic CKD by the Food and Drug Administration after successful phase III studies (5–7). Despite these advances, the residual risk of CKD progression and kidney failure remain high due to several factors, including underlying pathomechanistic heterogeneity (8, 9). Furthermore, patients have differential responses to treatments, highlighting the clinical importance of understanding disease heterogeneity. One approach to understanding disease heterogeneity is by characterizing the underlying differences in molecular phenotypes (8, 10). This approach has focused on well-characterized molecular phenotypes along a single -omics data type (e.g., transcriptomics). Additionally, multiple -omics data types can be integrated, in aggregate, to characterize molecular clusters. Multi-omics data integration has many applications, including biomarker discovery (11), drug and target discovery (12), drug repurposing (13), and patient stratification (14). These integration and systems biology approaches have been used to explore molecular pathophysiology in other diseases, including cancer (15), type 2 diabetes (16), osteoarthritis (17), Alzheimer disease (18), systemic lupus erythematosus (19), inflammatory bowel disease (20), and in rare diseases (21). We can now apply these approaches to CKD.

In molecular profiling studies of CKD, a significant challenge lies in comprehensively exploring the interrelationships across diverse high-dimension -omics data types to molecularly define or classify patients. Advanced statistical and machine-learning techniques, particularly multivariate- and Bayesian-based methods, have demonstrated superior performance in low to moderate sample sizes (22, 23). Here, we leverage 2 validated computational algorithms for integrating multiple -omics datasets, multi-omics factor analysis (MOFA) and data integration analysis for biomarker discovery using latent components (DIABLO). MOFA, an unsupervised algorithm, identifies sources of disease-associated variation by generating computed factors (24, 25). DIABLO is a supervised method that focuses on uncovering disease-associated multi-omic patterns (26). Notably, both MOFA and DIABLO were specifically validated for chronic diseases and exhibited robust performance even with small sample sizes (26, 27). By harnessing both these validated computational algorithms, we hypothesized that the challenges posed by the complexity of CKD could be overcome to gain insights into disease-associated variation and multi-omic patterns. This study applied this nuanced and innovative approach to molecularly define and classify disease biology in the context of CKD.

Longitudinal and multi-omics data from the Clinical Phenotyping and Resource Biobank Core (C-PROBE) cohort were used for this study. C-PROBE is a multisite cohort designed to accelerate translational research in kidney disease (28). The availability of multi-omic profiles for participants in C-PROBE facilitated a comprehensive evaluation of the effectiveness of MOFA and DIABLO. The selection of urine and plasma proteomic, kidney transcriptomic, and metabolomic data, coupled with longitudinal information, was deliberately designed to strategically capture diverse facets of molecular information associated with CKD progression in this cohort. By encompassing this spectrum, our study was designed to capture a range of molecular events implicated in CKD progression.

## Results

**Figure 1:**
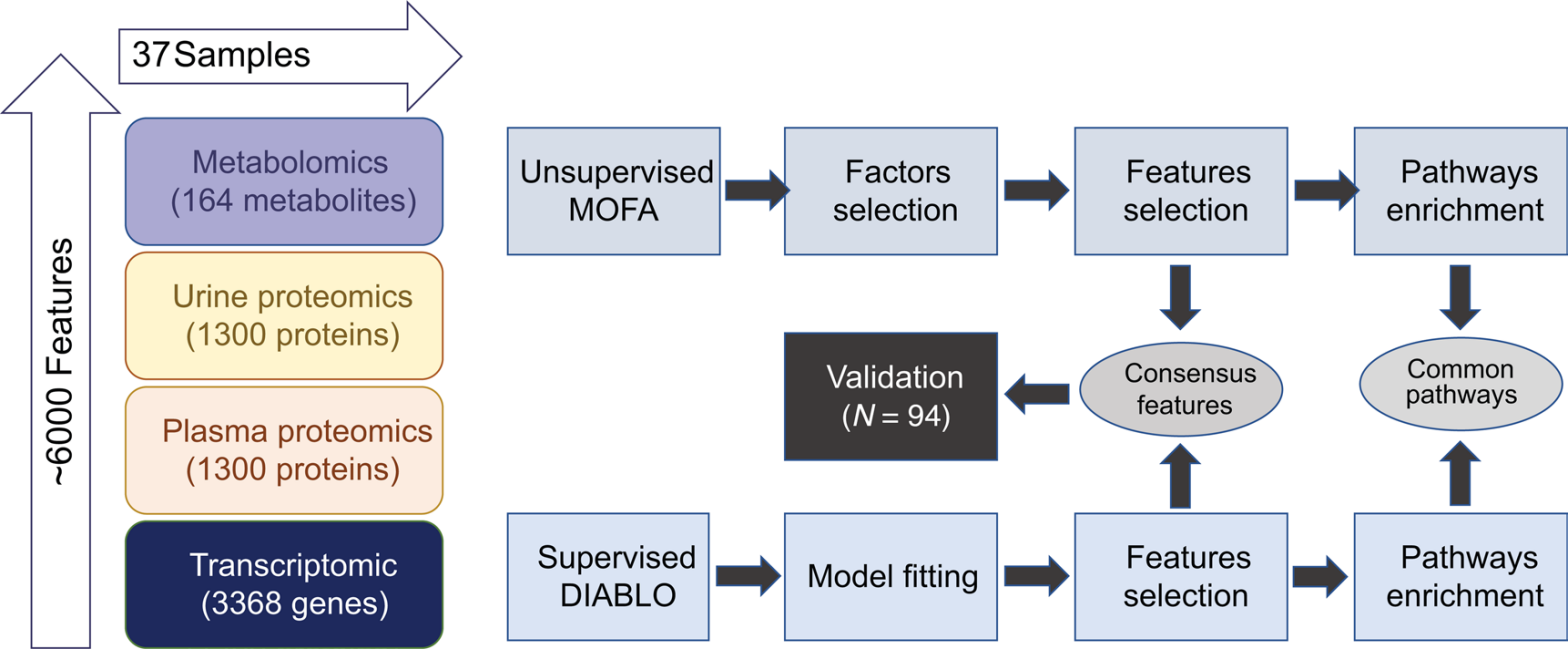
MOFA and DIABLO integrative approaches applied to the C-PROBE cohort. First step is training of the MOFA and DIABLO models. Second is the visualization of the variation and analysis of top-ranked features by both algorithms. Pathway analysis for top-ranked features is the third step. Validation of shared features in C-PROBE is the fourth step in this integrative approach.

We developed a comprehensive framework to integrate diverse data points (henceforth denoted as features) from metabolomics, urine proteomics, plasma proteomics, and tubulointerstitial transcriptomics from a discovery cohort 37 participants enrolled in the CKD C-PROBE cohort, coupled with longitudinal data using the 2 independent integration approaches, MOFA and DIABLO (Figure 1 and Supplemental Figure 1; supplemental material available online with this article; https://doi.org/10.1172/jci.insight.186070DS1). An additional 94 participants from C-PROBE that were independent of the discovery cohort were used to validate select findings from the MOFA and DIABLO integration.

To determine whether this framework might be broadly applicable to CKD, we used discovery and validation cohorts of varying clinical and histopathological makeup. Baseline clinical characteristics are provided for participants included in the discovery and validation datasets, including those who reached the composite CKD endpoint, in Table 1. The discovery cohort consisted of patients diagnosed with nondiabetic glomerular diseases with an average age of 39 years, a 5-year follow-up period, an estimated glomerular filtration rate (eGFR) average of 74 mL/min/1.73 m^2^, and a urinary albumin to creatinine ratio (uACR) average of 1.89 mg/mg. Participants in the validation cohort were older, predominantly diagnosed with DKD, had lower average eGFR (58 mL/min/1.73 m^2^), and lower uACR (0.69 mg/mg) in comparison with the discovery cohort, with 33 participants reaching the composite endpoint (Table 1). The observed differences in eGFR and uACR between the cohorts may be attributed to both age and distinct disease etiologies.

The number of features in the transcriptomics data (16,840 features) was more than an order of magnitude larger than the number of features in the next largest platform (1,301 urine and plasma features) and 2 orders of magnitude greater than the smallest platform included (164 metabolomic features). To normalize the data space to comparable dimensionality across data types, the top 20% most variable individual gene expression profiles across the 37 samples were retained. This resulted in 3,368 gene expression profiles as input features. The analysis pipelines for the 2 distinct integration algorithms employed are summarized in Supplemental Figure 2. MOFA, depicted in Supplemental Figure 2A, integrates multiple -omics datasets, capturing their complex interrelationships. DIABLO, illustrated in Supplemental Figure 2B, identifies shared variation across datasets through multivariate analysis, providing a comprehensive view of common molecular patterns.

### Unsupervised data integration using MOFA identified key disease-associated mechanisms.

**Figure 2:**
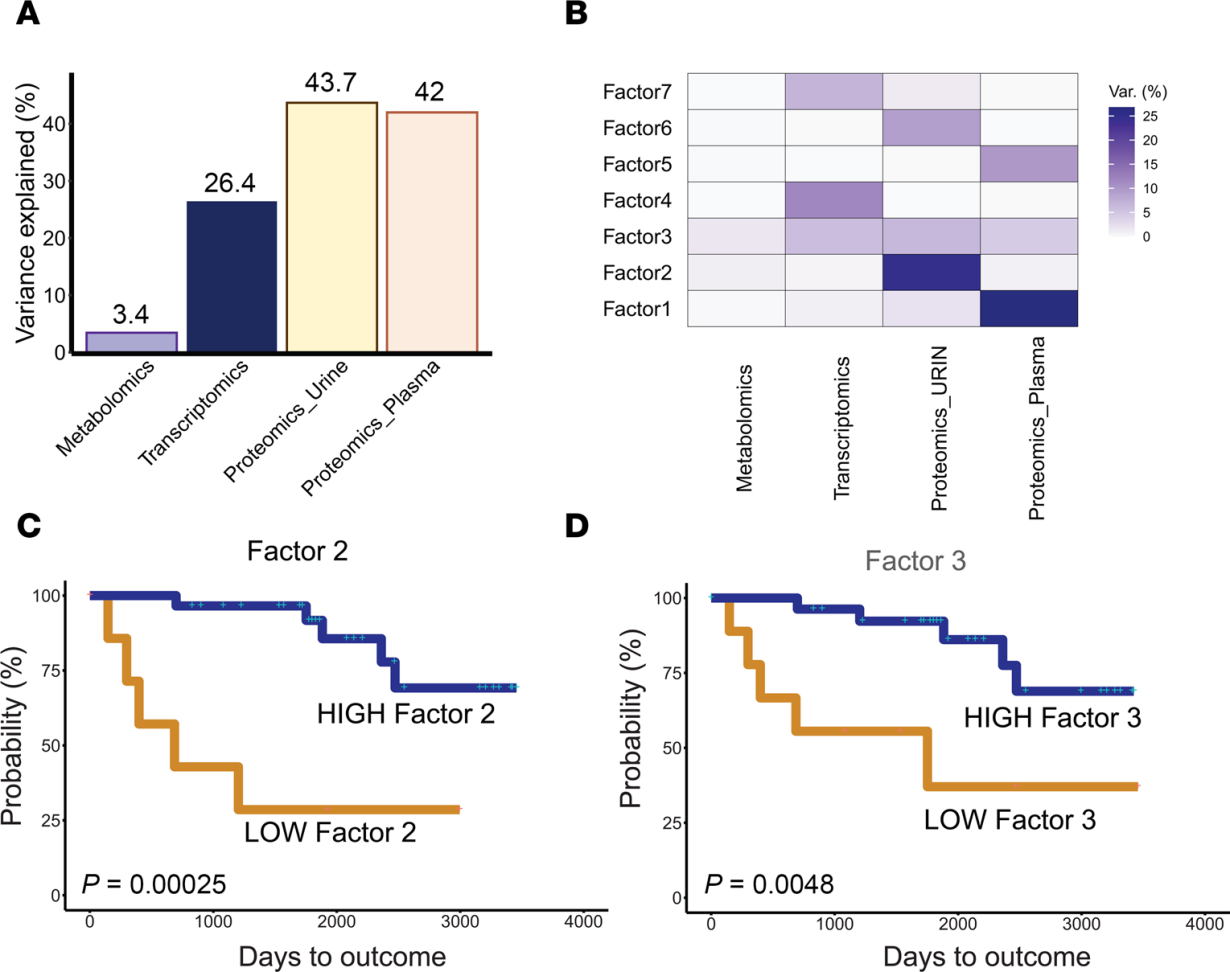
Factors from MOFA model. (**A**) Total percentage of variance explained by MOFA factors. (**B**) Data variance explained by each MOFA factor. (**C**) Kaplan-Meier (KM) survival curve using the value of MOFA Factor 2 reaching composite endpoint. (**D**) KM survival curve using the value of MOFA Factor 3 reaching composite endpoint. Log-rank test was used to determine significant differences in KM curves.

The first step in the unsupervised MOFA analysis was to reduce the dimensionality of the -omics data from 6,134 input features into uncorrelated and independent factors. Based on MOFA guidelines for factor selection with the given dataset dimensionality (24), we identified 7 independent factors (outlined by *K* = 7) from the 6,134 input features (Supplemental Figure 3A). With 7 factors the model explains 42% of the variation in the plasma proteomic data, 43.7% of the variation in the urine proteomic data, 26.4% of transcriptomic data, and 3.4% of metabolomic data (Figure 2A). MOFA Factors 1 and 5 explained most of the variance in plasma proteomics, Factors 2 and 6 explained most of the variance in urine proteomics, Factor 3 explained variance across all data types, while Factors 4 and 7 mostly explained variance in transcriptomic data (Figure 2B).

Because MOFA is a framework for unsupervised discovery of inherent variability in a given dataset, we next sought to prioritize factors for further investigation by asking whether any MOFA factors identified were associated with outcomes. Participants were grouped into either high or low factor expression categories based on optimal cut points derived from survival analysis, which assessed their risk of reaching the composite kidney endpoint for disease progression (40% loss of eGFR or kidney failure); significance was determined by log-rank test. Lower levels of Factor 2 and Factor 3 were significantly associated (*P* = 0.00001 and *P* = 0.00048, respectively) with CKD progression as shown in Kaplan-Meier (KM) curves (Figure 2, C and D, and Supplemental Figure 3, B–F). As noted above, Factor 2 was explained by variance in urine proteomic profiles, while Factor 3 was explained by variance across multiple -omics types (Figure 2B).

**Figure 3:**
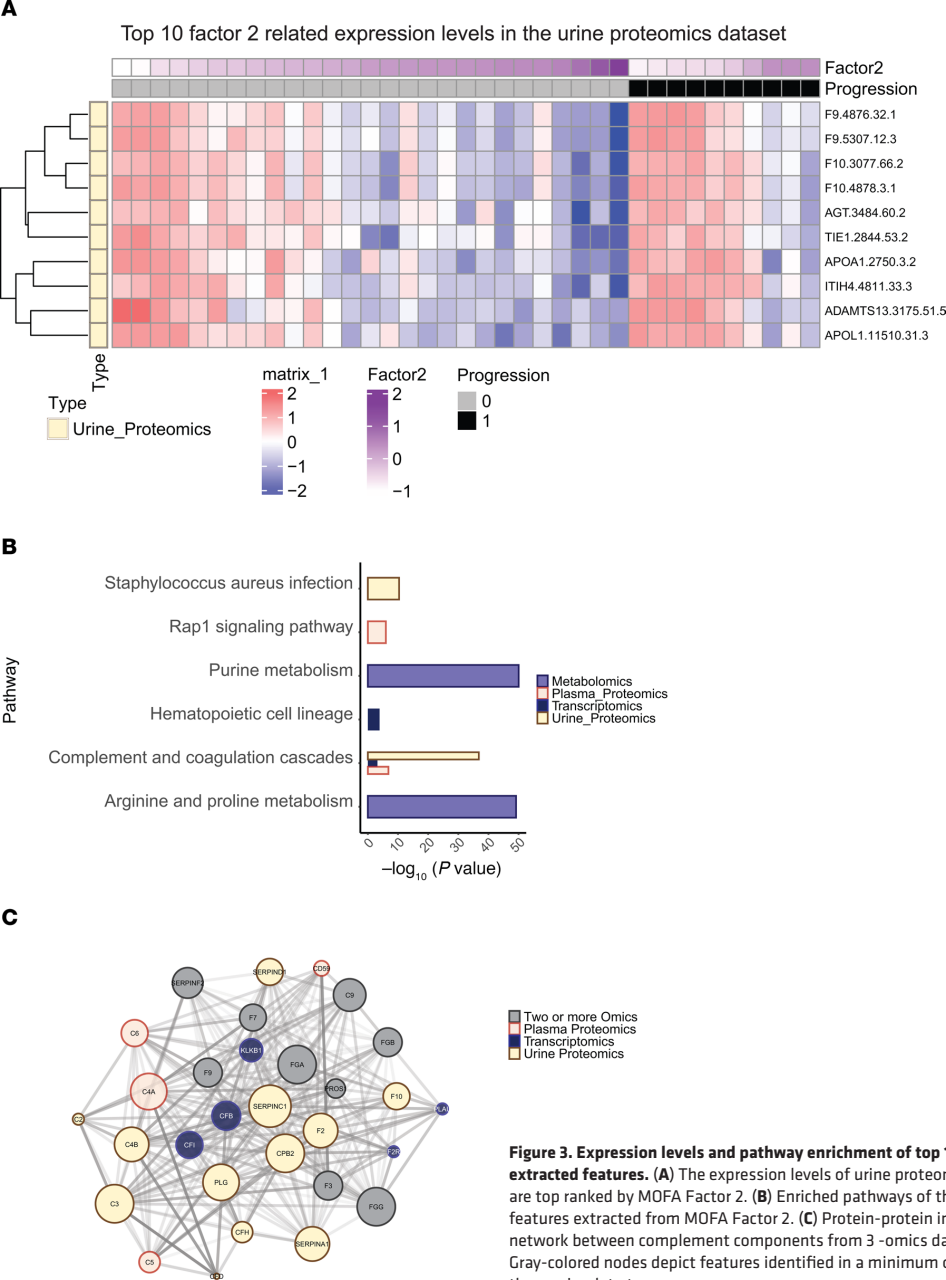
Expression levels and pathway enrichment of top 10 MOFA extracted features. (**A**) The expression levels of urine proteomics that are top ranked by MOFA Factor 2. (**B**) Enriched pathways of the top 100 features extracted from MOFA Factor 2. (**C**) Protein-protein interaction network between complement components from 3 -omics data types. Gray-colored nodes depict features identified in a minimum of any 2 of the -omics data types.

Given the associations with outcomes for Factors 2 and 3, we explored the contributing features to understand the underlying biology. Interestingly, many of the urine features, which explained the majority of variance in Factor 2, were assigned a negative weight by the MOFA model and were inversely correlated with Factor 2 expression (Figure 3A). As can be seen in the heatmap, urinary protein analytes F9, F10, APOL1, and AGT were among those contributing the most to Factor 2. Given that these proteins were inversely correlated with Factor 2, higher expression levels of these features are associated with worse outcomes. The top features from each -omics domain and weighting of all features contributing to Factor 2 are included in Supplemental Figure 4A and Supplemental Table 1, respectively. Similarly, many features contributing to Factor 3 were also inversely correlated with Factor 3 expression found in Supplemental Table 2 and depicted in Supplemental Figure 5A.

To characterize biology most associated with each factor, the top 100 features ranked by MOFA Factor 2 and Factor 3 were used for pathway enrichment analysis. The complement and coagulation cascades pathway was enriched across the 3 -omics data types (Figure 3B and Supplemental Figure 4, B–D) in Factor 2. Enriched complement components in urine proteomics, intrarenal transcript expression, and plasma proteomics were highly connected in a protein-protein interaction network derived from the STRING (29) database (Figure 3C). For MOFA Factor 3, the pathway enrichment analysis revealed the enrichment of the cytokine–cytokine receptor interaction pathway across 3 -omics data types (Supplemental Figure 5, B–E). Furthermore, the urine proteomics profiles derived from Factor 3 exhibited specific enrichment in the JAK/STAT signaling pathway (Supplemental Figure 5C). Notably, a number of additional shared pathways were identified from enrichment analysis of Factors 2 and 3, including the PI3K/Akt signaling pathway, MAPK signaling pathway, NF-κB signaling pathway, Rap1 signaling pathway, and axon guidance, suggesting the MOFA factors are independently capturing orthogonal features in CKD that are shared across multiple pathways. The primary features contributing to Factors 2 and 3 are included in Supplemental Tables 1 and 2, respectively.

### Supervised data integration using DIABLO identified key disease-associated mechanisms.

**Figure 4:**
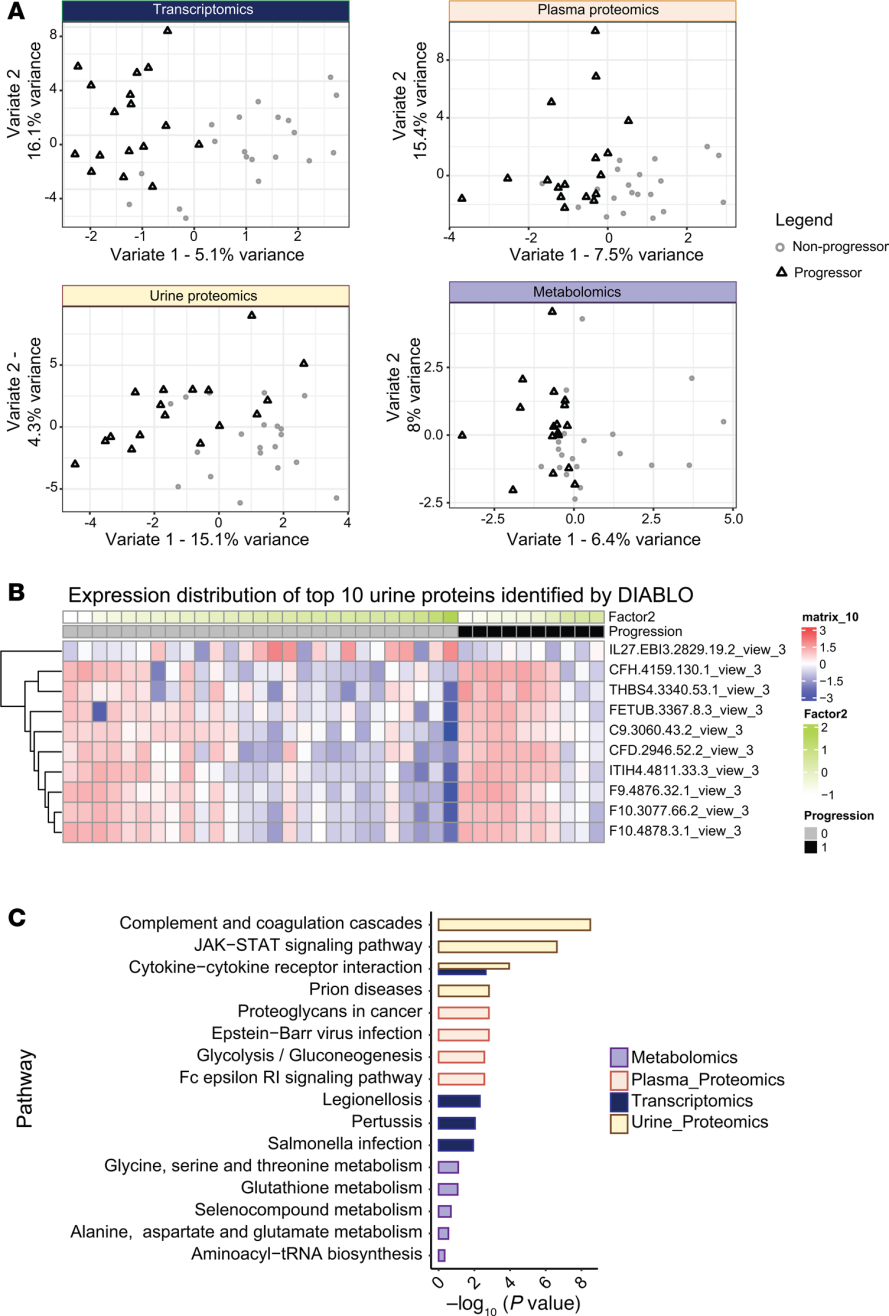
Top 10 features, expression levels, and enriched pathways using DIABLO. (**A**) Sparse partial least-squares discriminant analysis (sPLS-DA) plot for features identified by DIABLO. (**B**) Normalized expression levels of top 10 features identified by DIABLO in progressors and nonprogressors. (**C**) Pathway enrichment analysis for features identified by DIABLO model. KEGG pathway was the top pathway identified from RNA and proteins by DIABLO, while the lower panel, created using DIABLO, identified metabolites using MetaboAnalyst.

We used the same input data as MOFA (6,134 input features) in a supervised analysis of the data where the 37 participants were stratified into progressors versus nonprogressors. DIABLO identified 38 mRNA species, 24 plasma protein features, 34 urinary protein features, and 12 metabolites (overall 108 features) that discriminated progressors from nonprogressors. These features that are highly correlated (coexpressed) also captured the variance across each -omics block (Figure 4A). Transcriptomics-, plasma-, and urine proteomics–driven variance was 21.2%, 22.9%, and 19.4%, respectively, while metabolite variance was 12.4%. Supplemental Table 3 lists features from all 4 -omics data types that were selected by DIABLO. Figure 4B represents the overall expression distribution of the top 10 urinary proteins identified by DIABLO. The expression levels of the top 10 features from all 4 -omics data types, demonstrating the integrated nature of data selection in DIABLO, are depicted in Supplemental Figure 6. Figure 4C shows the canonical pathways enriched in these 108 features. It depicts complement and coagulation cascades and JAK/STAT signaling as the top enriched KEGG pathway terms from urinary proteins and cytokine–cytokine receptor interactions from transcripts and urinary proteins, while glycine, serine, and threonine metabolism are among the top enriched metabolic pathways.

### Combining results from MOFA and DIABLO.

**Figure 5:**
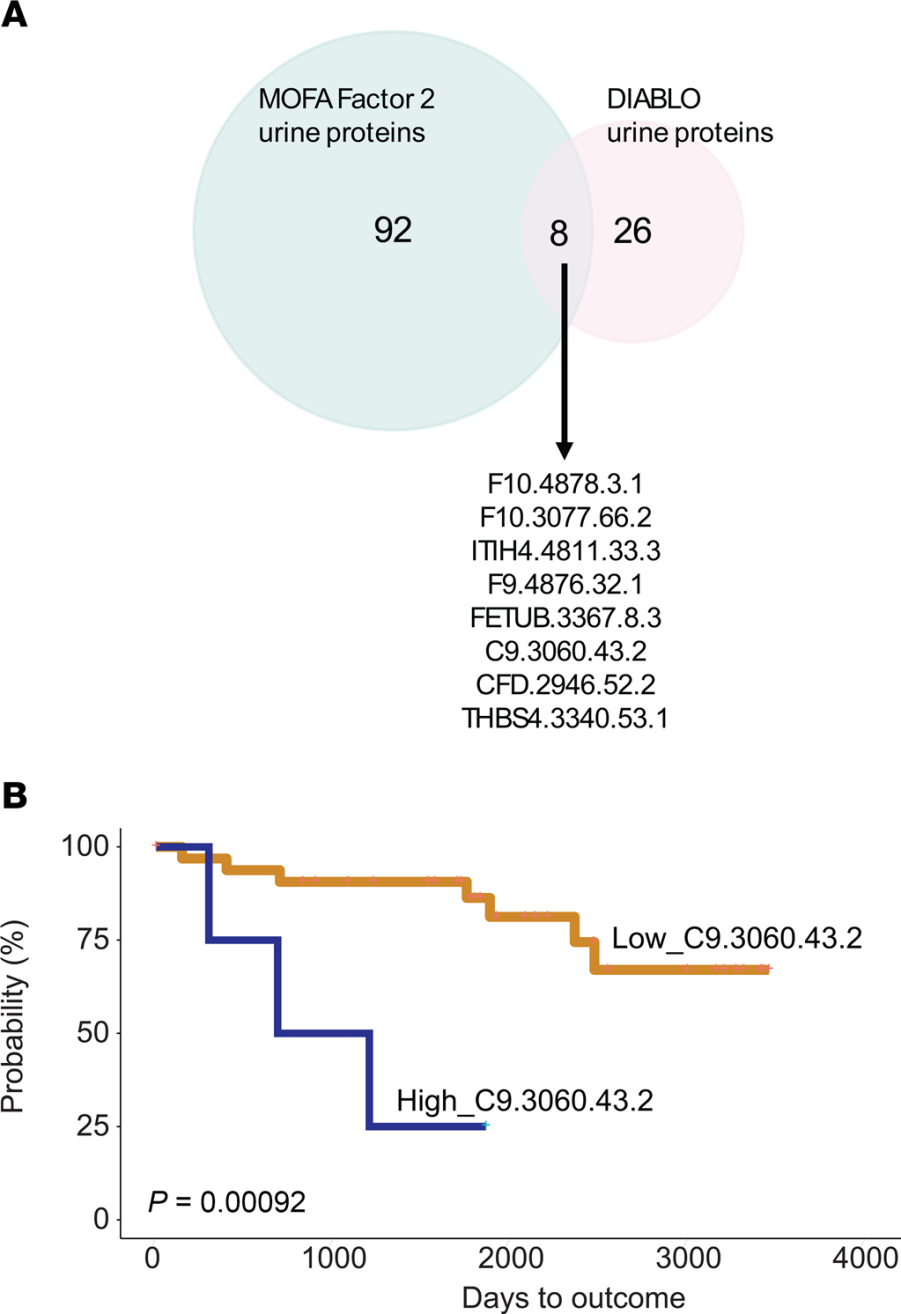
Consensus urinary proteins identified by MOFA and DIABLO. (**A**) Venn diagram of intersection between top 100 urinary proteins ranked by MOFA Factor 2 and 34 urinary proteins identified by DIABLO. (**B**) KM curve of 1 shared urinary protein, complement C9, identified by both methods depicting higher concentration is associated with increased risk of progression to composite endpoint in the validation cohort. Log-rank test was used to determine significant differences in KM curves.

We discovered that unsupervised MOFA Factor 2 (urine selective), MOFA Factor 3 (multi-omic driven), and supervised DIABLO results converged at the pathway level. Features contributing to MOFA Factor 2 and DIABLO resulted in enrichment for the complement and coagulation cascades pathway (Figure 3B and Figure 4C), while features contributing to MOFA Factor 3 and DIABLO resulted in enrichment for JAK/STAT and cytokine–cytokine receptor interactions pathways (Supplemental Figure 5B). This indicated that common features contributing to Factor 2 and Factor 3 from MOFA and features identified from DIABLO could be used to identify high-confidence findings in the urine. The intersect of 8 urinary protein probes, corresponding to 7 unique proteins, between the MOFA Factor 2 and DIABLO models (Figure 5A, details in Table 2) were significant (hypergeometric *P* < 0.003).

While MOFA selects factors independently of any outcome, DIABLO selects individual features based on their direct association with a desired endpoint. This targeted approach by DIABLO is evident in the significant associations observed between higher levels of specific urinary proteins and CKD progression. As shown in Table 2, higher levels of urinary proteins were significantly associated with CKD progression, as evidenced by the KM survival curves, with significant differences evaluated using the log-rank test (*P* < 0.05). An example KM curve is presented for complement C9 (Figure 5B). Additionally, the *P* values from the KM model of other shared urinary proteins are provided in Table 2.

MOFA Factor 3 (Supplemental Figure 5, C–E) and DIABLO (Figure 4C) jointly highlighted the JAK/STAT pathway and cytokine–cytokine receptor interaction pathway. In the cytokine pathway, MOFA Factor 3 and DIABLO shared enrichment of 2 transcripts (CXCL6, CXCL1) and 1 plasma protein (CCL23). In the JAK/STAT pathway, only 1 urinary protein (PIAS4) was enriched in both DIABLO and MOFA Factor 3 (Supplemental Table 4). A full intersection of pathways identified by MOFA Factor 2, MOFA Factor 3, and DIABLO are presented in Supplemental Table 5), which includes PI3K/AKT, NF-κB, and MAPK signaling pathways shared across MOFA factors and/or DIABLO. There are also shared infection-related pathways, which may be indicative of Toll-like receptor 2 (TLR2) and TLR4 activation.

As these urinary proteins had also been measured in other, independent samples in C-PROBE, we verified the identified proteins from the discovery cohort using the urine proteomics profiles from 94 independent C-PROBE participants in the validation cohort (Table 1) for association with CKD progression. Transcriptomic profiles were not available for these participants at the time of the study.

### Significance of shared urine profiles after adjusting for clinical variables.

**Figure 6:**
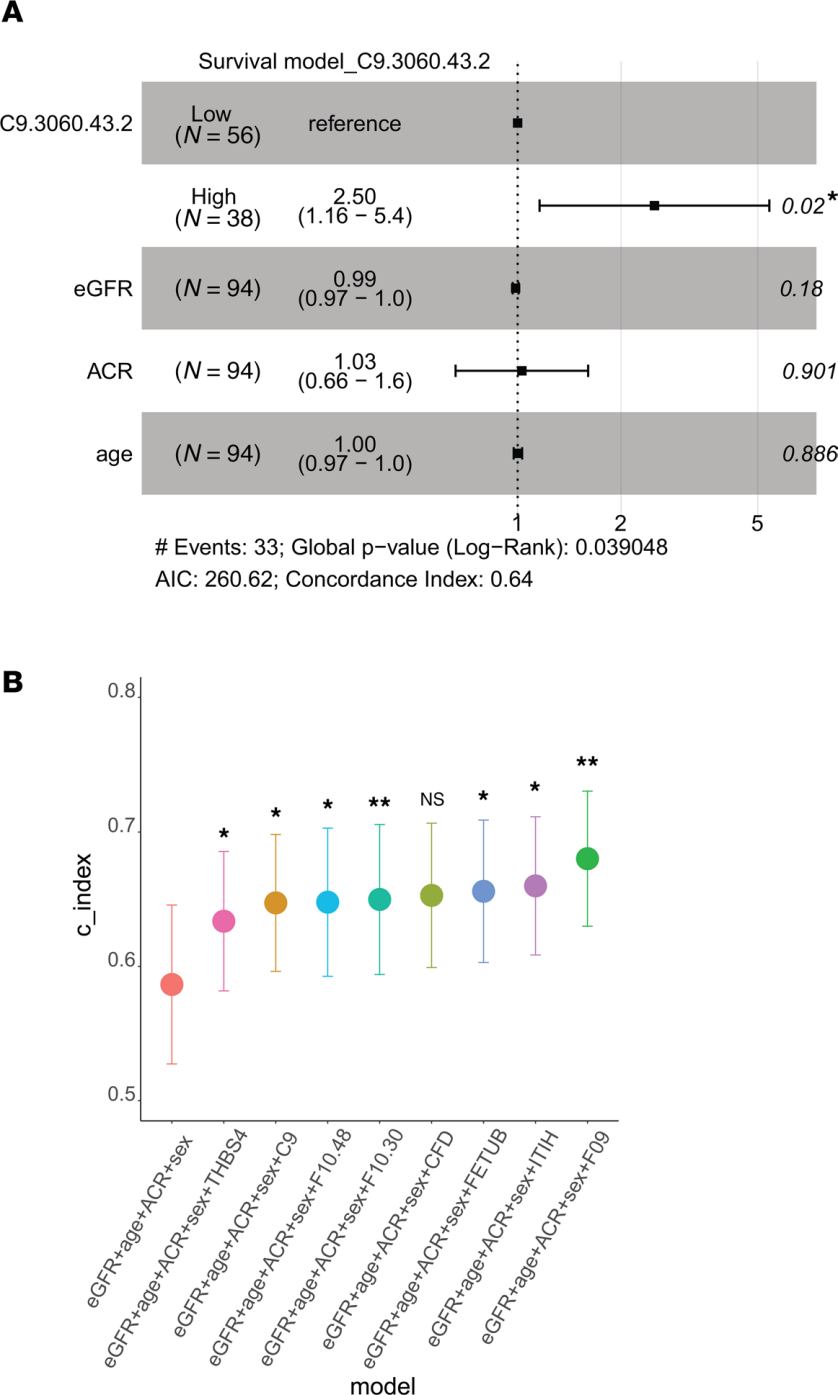
Validation of MOFA and DIABLO shared urinary proteins in independent C-PROBE samples. (**A**) CoxPH survival model, representing the composite endpoint outcome, for one of the shared urinary proteins, complement C9, adjusted for baseline estimated glomerular filtration rate (eGFR), sex, and age at first visit. (**B**) C-index value of basic model (eGFR, age, sex, and albumin to creatinine ratio [ACR]) compared with models built by adding 8 shared urinary proteins to the basic model. The c-index or c-statistic is the most frequently used evaluation metric of survival models. The c-index value ranges from 0 (perfectly discordant) to 1 (perfectly concordant), and a c-index of 0.5 suggests that the model’s predictions are no better than random chance. *P < 0.05 based on the likelihood ratio test to compare the goodness of fit of the urinary protein model (eGFR + age + sex + ACR + urinary protein) and the basic model (eGFR + age + sex + ACR).

As previously indicated, in all but one case, urinary protein features improved on the ability to predict the composite endpoint over the base model comprising eGFR, age, sex, and ACR. The Cox proportional hazards (CoxPH) model for C9 is shown (Figure 6A). Additionally, the *P* values from the CoxPH model of other shared urinary proteins are provided in Table 2. Moreover, adding these shared urinary proteins to the base model improved the model performance in predicting the composite endpoint, as shown by the increase in c-index values (Figure 6B).

### Kidney tissue transcriptomics and proteomics corroborate urinary proteomic signatures for complement pathway.

We also utilized the transcriptomics data from an independent cohort (European Renal cDNA Bank, ERCB) to identify genes differentially expressed in tubulointerstitial and glomerular compartments between CKD and living donor patients. As can be seen in Supplemental Figure 7, many genes annotated in the complement pathway were also upregulated in CKD compared with living donor patients in the tubulointerstitium (Supplemental Figure 7A) and glomerulus (Supplemental Figure 7B). Many of these complement pathway genes were negatively correlated with eGFR in both the tubulointerstitial and glomerular kidney compartments (Supplemental Figure 7, C and D). Gene activation of the complement pathway within the kidney is consistent with the broader, systemic complement/coagulation cascade that was reflected in the urine.

Lastly, we analyzed a publicly available kidney tissue proteomic DKD dataset from an independent cohort (30) to assess whether proteins identified by the intersect of MOFA and DIABLO were also elevated in tissue SOMAscan profiling. We took the 7 progression-associated urinary proteins identified in our analysis and hypothesized that SOMAscan probes representing these proteins would be elevated in DKD samples from kidney tissue. Nine probes were identified in kidney tissue that passed SOMAscan QC parameters that corresponded to the 7 urinary proteins. Five of the 7 progression-associated urinary proteins (CFD, C9, F9, FETUB, F10) had corresponding probes in kidney tissue SOMAscan data that were elevated in DKD compared with controls (*P* < 0.05) (Supplemental Table 6). These findings are consistent with elevated tissue-level gene expression of complement pathway genes in CKD and corroborate our urine proteomic findings.

## Discussion

In this work, we developed a framework for integrating 4 -omics data types from patients with CKD recruited to the C-PROBE cohort using 2 different integrative algorithms. The implementation of complementary MOFA and DIABLO computational methods allowed us to determine the combined effect of 4 -omics datasets on CKD progression. This combined computational approach identified the complement and coagulation cascades, cytokine–cytokine receptor interaction, and JAK/STAT signaling as key molecular mechanisms associated with CKD progression. We also identified enrichment of PI3K/AKT, NF-κB signaling, and MAPK signaling shared across MOFA factors and/or DIABLO. These pathways are involved in cell survival, aging, proliferation, metabolism, and inflammation, all hallmarks of CKD (31, 32). The innate immune pathway components, TLR2 and -4, were identified in the overlap of MOFA Factors 2, 3, and Diablo. The activation of this innate immune pathway in CKD is well documented. For example, in preclinical animal models, pharmacological targeting of the TLR4 pathway slowed kidney disease progression and found to be kidney protective. Inflammatory processes downstream of TLR2 and TLR4 converge on NF-κB activation (33), a key regulatory hub in CKD (34). Therefore, this approach identified known disease-related mechanisms.

We chose to take a multi-omics integration strategy in our systems biology approach to CKD progression for several reasons. In contrast with single -omics approaches, integration across different -omics platforms is less dependent on biases and potentially misleading signals arising from individual -omics data (35). CKD is complex, and multi-omics integration captures disease heterogeneity, which might be missed by focusing exclusively on any single -omics data type. For example, transcripts responding to the chronic disease state may not yet be captured in proteomic outputs or plasma proteomic profiles but can indicate pathway signaling and transcriptional activation in the kidney. The complexity of CKD arises from its multifactorial nature, involving various molecular pathways and contributing factors. While a single -omics dataset provides valuable insights into specific aspects of the disease, it may not capture the full spectrum of heterogeneity present in CKD. From a practical standpoint, the inclusion of multi-omics data and deep patient molecular phenotyping allowed us to study a relatively small (*n* = 37) patient cohort to extract biologically meaningful and statistically significant inferences.

Different technologies used for measurements of transcripts, protein, and metabolites, such as SOMAscan, Affymetrix GeneChip, RNA-seq, LC-MS, GC-MS, and NMR, have their own limitations, sources of error, and variability, which pose inherent analytical and interpretation challenges. For instance, SOMAscan measures native proteins in complex matrices by transforming each protein concentration into a corresponding SOMAmer reagent concentration, which is then quantified by standard DNA microarray techniques. On its own, this technology can have many sources of variability, such as nonspecificity, batch effects, sequence variation, and cross-reactivity, and, as with all proteomic profiling techniques, it requires independent validation through orthogonal techniques such as ELISA (36). Similarly, measuring metabolites is inherently challenging due to their reactivity, structural diversity, and broad concentration range. Metabolites’ concentrations have large variabilities due to different sample preparation methods, enzyme assays, and analytical platforms such as LC-MS, GC-MS, and NMR (37).

Both MOFA and DIABLO independently highlighted significant variability in urinary proteins among patients with kidney disease. It is important to note that DIABLO, being a supervised model, incorporated the labels of progressors and nonprogressors to identify features associated with a binary outcome label without considering the time to achieve the progression status. MOFA avoids this limitation as an unsupervised approach; however, the effects on progression were verified subsequently using both KM and CoxPH analyses on individual MOFA factors as well as on features most contributed to each outcome-associated factor shared with DIABLO. The key differences in modeling strategies, while limiting on their own, were complementary in nature, enabled prioritization, and are a strength in our overall analysis adding to the robustness of reported signals.

Urinary proteins within the complement/coagulation cascades were identified by both approaches. Several clinical biomarker studies have demonstrated that complement pathway components are elevated in urine and plasma samples in DKD and a complement signature is associated with kidney function decline (38–41). In addition to revealing complement and coagulation pathway signatures, both MOFA and DIABLO algorithms independently identified the JAK/STAT and cytokine signaling pathways. A phase II clinical trial of baricitinib, a JAK1/JAK2-selective small molecule inhibitor, was shown to have therapeutic efficacy in lowering albuminuria in DKD (42). Among the 4 -omics data layers fueling our study, MOFA and DIABLO independently identified a group of 8 common urinary proteins from the complement and coagulation pathways (Table 2), which demonstrated significant association with CKD progression as a group. Importantly, these urinary proteins were also significantly associated with progression in the C-PROBE validation cohort, and significantly improved model predictive performance for reaching the kidney composite endpoint. Urine has several advantages for inexpensive molecular diagnostics, including but not limited to it being a noninvasive biofluid that is routinely and easily collected during patient hospital visits. Given pathomechanistic heterogeneity in CKD, rapid molecular diagnostics for complement/coagulation pathway urinary signatures may serve as a relatively inexpensive method for CKD risk assessment. Our observations motivate further studies along these lines, as well as further elucidation of a potential causal role in CKD progression, with the goal of developing precision medicine therapies targeting these pathways. To the best of our knowledge, this is the first study that integrates 4 -omics data types profiled from the same patients in a longitudinal CKD cohort. It demonstrates that multi-omics data integration approaches can help overcome the limitations of small cohort/sample sizes in the field of kidney disease. While integration was successful at gleaning biological insight from different data sources, the study had a few limitations.

The major limitation is that the size of the discovery cohort was small. While we were able to detect differences explained by MOFA and DIABLO, our power to detect other more subtle changes in feature expression was not there. Additionally, MOFA factors only explained a small variance (3.4%) in metabolite profiles, which may be attributed to the relatively low dimensionality of metabolites compared with transcriptomic and proteomic profiles and the small sample size of the discovery cohort. Despite these limitations, metabolomic profiles contributed to MOFA Factor 2, and could unveil unique insights that might be overlooked if metabolomics were excluded. DIABLO identified a limited number of metabolites associated with CKD progression, reinforcing the importance of including metabolomics to capture comprehensive biological variations. The discovery cohort is heterogeneous for histopathological diagnoses, which adds to the underlying biological variability. The output of both MOFA and DIABLO highlighted urinary proteins, which seemingly undermines the notion that multifactor integration improves prediction. It is possible that urinary proteins may simply be better predictors of outcome than other -omics features; however, given the sample size and data availability limitations, this could not be investigated in the current study. The intersect of MOFA and DIABLO identified urinary SOMAscan probes representing proteins in the complement and coagulation pathways. Whether these represent cleavage products or whole proteins as predictive of progression is at this point undetermined. Improving transcriptomic selection by refining genes into targeted functional units, including but not limited to molecular signature classes (10), coexpression modules (43), or reference-based cell-selective expression profiles (44), may lead to better prediction from transcriptomic profiles. Relying on the union of unbiased (MOFA) and biased (DIABLO) could unintentionally introduce biases into the findings (45). One of the limitations of our study is the focus on tubulointerstitial transcriptomics without including glomerular transcriptomic profiles. This exclusion, dictated by sample availability and study design, limited our ability to compare molecular signatures across different renal compartments, potentially overlooking critical insights into CKD progression. However, previous research, including our own findings in the supplemental material and studies on rare kidney diseases (8, 46), highlights similarities in inflammatory signaling between the glomerular and tubulointerstitial compartments (8). Lastly, CKD patients in the validation cohort were significantly older, had lower eGFR at time of sample collection, had improved albuminuria, and over half of the patients were diagnosed with diabetic nephropathy compared with none in the discovery cohort. Despite these limitations, when adjusted for age, uACR, and eGFR, all but one of the combined markers identified by MOFA and DIABLO remained significantly associated with progression.

Taken together, we were able to demonstrate, as a pilot study, a successful multi-omics integration approach, despite sample size limitations. While the top mechanisms identified are known to CKD, we feel this approach will prove beneficial in the future to several large ongoing cohort studies in kidney diseases, to help identify crucial disease mechanisms. Extending observations beyond a 1-gene hypothesis, we implemented a multiple genes and proteins discovery framework that identified the complement and JAK/STAT pathways as shared signals between the approaches. The ability to identify these known CKD pathways does support the ability of this approach to identify relevant disease biology, which may be improved in larger studies. The findings from this study were aligned with prior knowledge of kidney pathophysiology and help establish a computational framework for future studies.

## Methods

### Sex as a biological variable.

This study included male and female participants enrolled in the C-PROBE cohort (Table 1). Sex was not considered as a biological variable in the discovery analysis or for statistical associations unless otherwise noted. CoxPH models reported in this manuscript included sex as a biological variable.

### Study population.

C-PROBE is a multiethnic, prospective observational cohort of patients with CKD stages I–IV, which collected clinical information and biospecimens at enrollment and yearly thereafter at 6 sites in the United States (28, 47). eGFR was calculated using the CKD-EPI equation (48, 49). Albuminuria was assessed by Albuwell Hu ELISA Kit (Ethos Biosciences). eGFR slopes were calculated using linear mixed-effects models. The C-PROBE cohort used in this study included participants with and without kidney biopsy transcriptomic profiles. Therefore, only the 37 participants with kidney biopsy–derived transcriptomic, urine and plasma proteomic, and urine metabolomic profiles were included in the discovery cohort. All the above 4 multimodal measurements were derived from the same samples.

Additionally, data from an independent set of 94 participants in C-PROBE, separate from the discovery cohort, were used to validate select findings from the MOFA and DIABLO integration. This group was specifically selected based on the availability of urine proteomic profiles. Clinical characteristics of the 94 independent samples from the validation set can be found in Table 1.

### Transcriptomics data.

Transcriptomics data were derived from microdissected tubulointerstitial components of human kidney biopsies prospectively procured for molecular analysis, using Affymetrix GeneChip microarrays as previously published (47). The CEL files were normalized by the Robust MultiArray method and annotated with the Human Entrez Genes custom cdf version 10 (http://brainarray.mbni.med.umich.edu). Normalized expression value data were log_2_ transformed. The CEL files are available in the NCBI Gene Expression Omnibus (www.ncbi.nlm.nih.gov/geo/) under GSE69438.

### Urinary metabolomics data.

Detailed metabolomics analyses were performed using targeted hydrophilic interaction liquid chromatography coupled with tandem mass spectrometry (HILIC-LC-MS/MS). Samples were thawed on ice, after which 300 μL of a prechilled mixture of acetonitrile/isopropanol/water (3:3:2 v/v) was added to 50 μL of each urine sample. The samples were mixed thoroughly and incubated at –20°C overnight. The samples were then centrifuged for 15 minutes at 3,200*g* and 4°C, and the supernatants were removed for LC-MS/MS analysis. A pooled QC sample was created by taking an aliquot of the same volume from all the extracted samples in the study. This pooled sample was then serially diluted to form a standard curve. The study samples were then further diluted 1:1 with the same prechilled acetonitrile/isopropanol/water mixture prior to analysis. LC-MS/MS was performed using a Shimadzu Nexera UPLC system coupled with a SCIEX Triple Quadrupole 6500+ mass spectrometer. HILIC-LC separation was achieved using an apHera NH2 column (15 cm × 2 mm, 5 μm particle size, Supelco Analytical) with an apHera NH2 guard column (1 cm × 2 mm, 5 μm particle size, Supelco Analytical). The mobile phases were 50 mM ammonium bicarbonate (pH 9.5, adjusted with ammonium hydroxide) (A) and acetonitrile (B). After a 3-minute isocratic run at 90% B, gradients to 85%, 75%, 45%, 30%, and 2% B were concluded at 3, 11.5, 15, 20, and 21 minutes, respectively. A gradient to 90% B was completed at 23.5 minutes and was followed by a 4-minute column equilibration. The flow rate started at 0.25 mL/min and increased to 0.35 mL/min at 20 minutes and 0.4 mL/min at 21 minutes and then went back to 0.25 mL/min at the end of the equilibration. Positive and negative scheduled MRM transitions were monitored for a total of 215 metabolites that were used as input features into the analysis. The metabolite peaks were integrated, and the AUCs and relative intensities of the metabolites were calculated using SCIEX MultiQuant 3.0.2 software.

### Urine and plasma proteomics data.

SOMAmer-based proteomic assays were performed on both plasma and urine samples using the SOMAscan 1.3K Plasma Kit and SOMAscan 1.3K Cells and Tissue Kit, respectively, following standard experimental and data analysis protocols (50). These assays were carried out by the Genome Technology Access Center at the McDonnell Genome Institute at Washington University School of Medicine. Urine features were normalized to urinary creatinine levels. Proteomic feature signals from plasma and normalized urine were used as input features for the analysis.

### Filtration and normalization.

For transcriptomics data, we retained transcripts with the highest variance (top 20%). Each -omics data type was normalized independently of other -omics data types. Data normalization was carried out by centering and scaling data in R (https://www.r-project.org/).

### MOFA.

We used MOFA model version 2.1.1 (https://github.com/bioFAM/MOFA2) to integrate C-PROBE multi-omics data sets in an unsupervised fashion. MOFA is suited to a cohort with a small sample size as in our case and it is an unsupervised approach to avoid model overfitting. MOFA is a versatile generalization of the principal components analysis that reduces the dimension of the -omics data into a few latent factors. These latent factors capture the source of the variation in the data and represent the linear combination of features from all 4 -omics data types. For downstream analysis, we used the MOFA factors that explained the data variability across most data modalities and were associated with disease progression. We used all MOFA default parameters for training. We used ggplot R package v2_3.3.2 and pheatmap version 1.0.12. for visualization.

### Survival analysis.

We used survival R package version 3.2-3. We used the ggsurvplot function from the survival R package to plot the KM survival curves and ggforest function from survminer R package version 0.4.8 to generate the Forest plot for the CoxPH model. We used the surv_cutpoint function to calculate the optimal cut points for the KM curves. We used the lrtest function from the lmtest R package to perform the likelihood ratio test to compare the goodness of fit of 2 nested survival models.

### Composite kidney survival endpoint used to define CKD progression.

The C-PROBE cohort is a longitudinal cohort with a 5-year median follow-up. Composite endpoint was defined as kidney failure or 40% reduction in eGFR from baseline. This criterion was used to create the binary categories of patients reaching the endpoint (median follow-up time 950 ± 870 days) termed progressors, and those who did not (median follow-up time 1,950 ± 923 days), termed nonprogressors for subsequent multi-omics analysis for disease-associated markers.

### DIABLO.

We used mixOmics R package version mixOmics_6.15.1 for the entire analysis (51). Normalized data across each -omics type were used as input for supervised DIABLO. Overall, 6,000 features from across all 4 -omics types were used for analysis with DIABLO. The tune.block.spslda (51) function was used to identify the optimal number of supervised features across each -omics type. A total of 28,561 models were fitted for each component and each *nrepeat*, using the centroid.dist parameter with a 5-fold cross validation to identify the optimal number of features across mRNA, protein, and metabolomics that are used for subsequent integration analysis downstream. Finally, these supervised feature numbers were fitted to block.splsda (51) with a weighted design matrix of 10% contribution across -omics types that can discriminate and explain the phenotypic variance. The *nComponents* parameter used was 2 in this analysis. The block.splsda function basically performs a supervised integration of sparse heterogeneous -omics datasets to identify coexpressed features across each -omics type that are highly correlated in a latent space and still able to capture enough variance to project the difference between the nonprogressor and progressor group in the C-PROBE discovery cohort, as defined using the aforementioned descriptions. The assumption of DIABLO is based on a pairwise linear combination of factors that reduces dimensionality of the data, captures biological variability, and identifies correlated (coexpressed) molecular events associated with our categorical phenotypic outcome. DIABLO basically decomposes the larger data matrices across each -omics type into smaller loading vectors and discriminating components (*nComponent*) to maximize the covariance between a linear combination of variables from each -omics type and categorical composite endpoint.

### ERCB transcriptomic profiles.

The ERCB is a European multicenter CKD study established to collect kidney biopsy tissue for gene expression analysis at the time of a clinically indicated biopsy. These data have been published and described elsewhere (10, 46) and deposited into the GEO under the accession numbers GSE104954 (tubulointerstitium) and GSE104948 (glomerulus). Briefly, probe sets were annotated to Entrez genes IDs using custom gene set probe definitions from Brainarray v19 (http://brainarray.mbni.med.umich.edu/Brainarray/Database/CustomCDF/). Arrays were RMA normalized using probe sets common to U133A and U133 Plus 2.0 and batch corrected using COMBAT (https://rdrr.io/bioc/sva/man/ComBat.html). Differential expression analysis was performed on the resulting expression data using the significance analysis of microarrays (SAM) analysis method in the Tigr MeV program (https://sourceforge.net/projects/mev-tm4/).

### Tissue proteomic profiles.

Publicly available protein probe data from DKD and control human kidney tissue samples (30) were extracted (https://data.mendeley.com/datasets/83k89shdx5/1) and log_2_ transformed. Validation of 7 progression-associated urinary proteins identified from the convergence of MOFA and DIABLO was determined using a 1-tailed *t* test was used to determine significance.

### Pathway enrichment.

Pathway enrichment analysis for the transcripts and proteins identified by DIABLO and MOFA were done using the Enrichr (52–54) (plus additional references) tool and KEGG database (55–57), while the MetaboAnalyst v6.0 (58, 59) web tool was used for metabolomics enrichment analysis.

### Statistics.

Associations between a binary category and a continuous variable were evaluated by *t* test, while ANOVA was utilized for variables with more than 2 categories. The χ^2^ test was applied for the analysis of associations between 2 categorical variables. A significance threshold of *P* less than 0.05 was considered for all tests.

### Study approval.

The C-PROBE study was approved by the University of Michigan Institutional Review Board (HUM 00020938). All participants provided written, informed consent at enrollment.

### Data availability.

The transcriptomic data supporting the findings of this study are available in the public domain in the NCBI GEO repository under accession IDs GSE69438 (C-PROBE tubulointerstitial profiles), GSE104954 (ERCB tubulointerstitial profiles), and GSE104948 (ERCB glomerular profiles). Access to the metabolite data and SomaLogic SOMAscan affinity-based aptamer proteomics data can be requested from the consortium via an ancillary application at https://kidneycenter.med.umich.edu/clinical-phenotyping-resource-biobank-core See the Supporting Data Values file for raw values associated with the main manuscript and supplemental figures.

### Code availability.

The code used to train the MOFA model is included in the supplemental material.

## Author contributions

The 2 first coauthors, FA and VD, each made unique and critical contributions to this manuscript and agreed on the order of authorship based on the lead taken in coordinating manuscript development. FA, VD, SE, VN, DF, MK, WJ, and AK designed the study. MK, MB, SP, WJ, and AK acquired funding for the study. WJ and MB led the cohort studies. FA, VD, DF, VN, and WJ acquired or generated the data. FA, VD, and DF analyzed the data. FA, VD, AM, ABD, FCB, MK, AK, and SE wrote the manuscript. MK, AM, ABD, TS, DFR, EM, KLD, YC, WJ, FCB, and SE provided scientific guidance and insights. All authors reviewed, edited, and approved the manuscript.

## Supplementary Material

Supplemental data

Supplemental table 1

Supplemental table 2

Supplemental table 3

Supplemental table 4

Supplemental table 5

Supplemental table 6

Supporting data values

## Figures and Tables

**Table 1:**
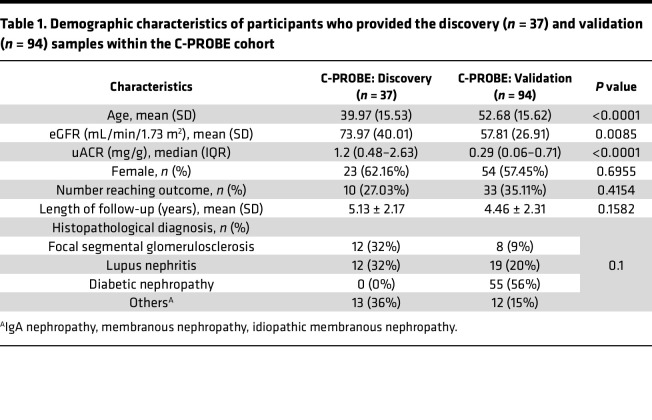
Demographic characteristics of participants who provided the discovery (*n* = 37) and validation (*n* = 94) samples within the C-PROBE cohort

**Table 2:**
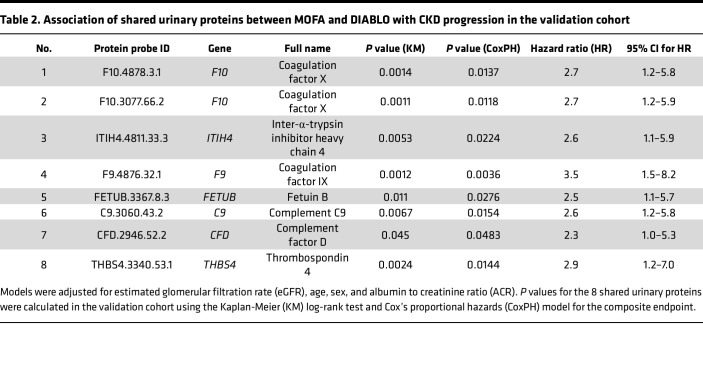
Association of shared urinary proteins between MOFA and DIABLO with CKD progression in the validation cohort

## Version 1 03/10/2025

Electronic publication
