# Supplementary material for: Leveraging complementary multi-omics data integration methods for mechanistic insights in kidney diseases
Source: JCI Insight. 2025 Mar 10;10(5):e186070. doi: 10.1172/jci.insight.186070 (PMC11949029; doi:10.1172/jci.insight.186070)
Supplement: Supplemental data [file jciinsight-10-186070-s189.pdf]

List of Supplementary Materials

R script for training of MOFA model

Supplementary Figures

Supplementary Figure S1. Intersection of Omics data types.

Supplementary Figure S2. Detailed whole pipeline of MOFA and DIABLO

Supplementary Figure S3. MOFA factors and its association with disease progression

Supplementary Figure S4. Top features ranked by MOFA for factor 2 and corresponding enriched pathways.

Supplementary Figures S5. Top features ranked by MOFA for factor 3 and corresponding enriched pathways.

Supplementary Figure S6. Top features selected by DAIBLO for factor 2.

Supplementary Figure S7. Pathway of complement and coagulation cascade KEGG pathway.

Supplementary Tables

Supplementary Table S1. MOFA features extracted from MOFA factor 2

Supplementary Table S2. MOFA features extracted from MOFA factor 3

Supplementary Table S3. A list of DIABLO features

Supplementary Table S4. Enriched Molecular Features Across Complement and Coagulation, Cytokine-Cytokine Receptor Interaction, and JAK-STAT Pathways

Supplementary Table S5. Pathways Common to DIABLO, Factor 2, and Factor 3 Among Top Ranked Urine and plasma Proteins and genes

Supplementary Table S6. Expression of MOFA and DIABLO progression-associated urine proteins in kidney tissue proteomic profiles

R script for training of MOFA model

```
# Load necessary libraries
library(MOFA2)
library(readxl)
library(ggplot2)
library(ComplexHeatmap)
library(survminer)

# Read normalized data files for each omics type
# All omics data were converted to z-score values separately
Tra <- read.csv("Trans_Normalized.csv", check.names = FALSE)  #
Reading transcriptomics data
Met <- read.csv("Meta_Normalized.csv", check.names = FALSE)   #
Reading metabolomics data
Ps <- read.csv("Ps_Normalized.csv", check.names = FALSE)      #
Reading plasma proteomics data
Urs <- read.csv("Urs_Normalized.csv", check.names = FALSE)    #
Reading urine proteomics data

# Prepare data list for MOFA model
data <- list(Met, Tra, Urs, Ps)

# Create MOFA object using the prepared data
MOFAobject <- create_mofa(data)

# Assign names to each view in the MOFA model
views_names(MOFAobject) <- c('Metabolomics', 'Transcriptomics',
'Proteomics_URIN', 'Proteomics_Plasma')

# Plot data overview to inspect the input data
plot_data_overview(MOFAobject)

# Set data options, here scaling the views to unit variance
data_opts <- get_default_data_options(MOFAobject)
data_opts$scale_views <- TRUE

# Set model options, specifying the number of factors
model_opts <- get_default_model_options(MOFAobject)
model_opts$num_factors <- 7  # Setting the number of factors to 7
based on earlier analysis

# Set training options
train_opts <- get_default_training_options(MOFAobject)

# Prepare the MOFA model with specified options
MOFAobject <- prepare_mofa(MOFAobject,
                           data_options = data_opts,
                           model_options = model_opts,
```

```
                             training_options = train_opts)

# Run the MOFA model
MOFAobject <- run_mofa(MOFAobject, outfile =
'./bestModel/MOFA2_FADHL_GD_37Samples_NG.hdf5')

# Plot the correlation matrix of the factors to inspect relationships
between them
plot_factor_cor(MOFAobject)

# Plot variance explained by each factor and by each view
plot_variance_explained(MOFAobject, x = "view", y = "factor", max_r2 =
20, plot_total = TRUE)[[1]]

# Extract and print the factor values to check the output
factors <- get_factors(MOFAobject,
                       factors = "all",
                       as.data.frame = TRUE)
head(factors)  # Display the first few rows of the factor values
```

Supplementary Figure S1

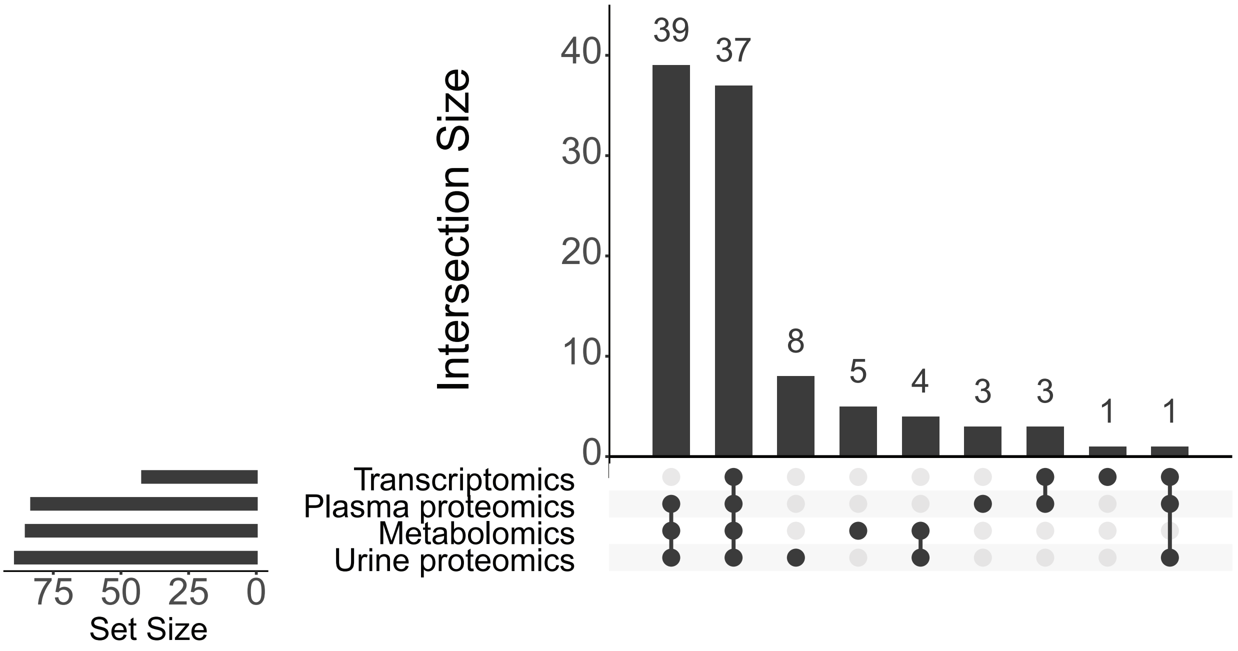

Supplementary Figure S1. **Intersection of Omics data types.** The UpsetR plot of the number of samples had complete four omics data including: metabolomics, proteomics and transcriptomics.

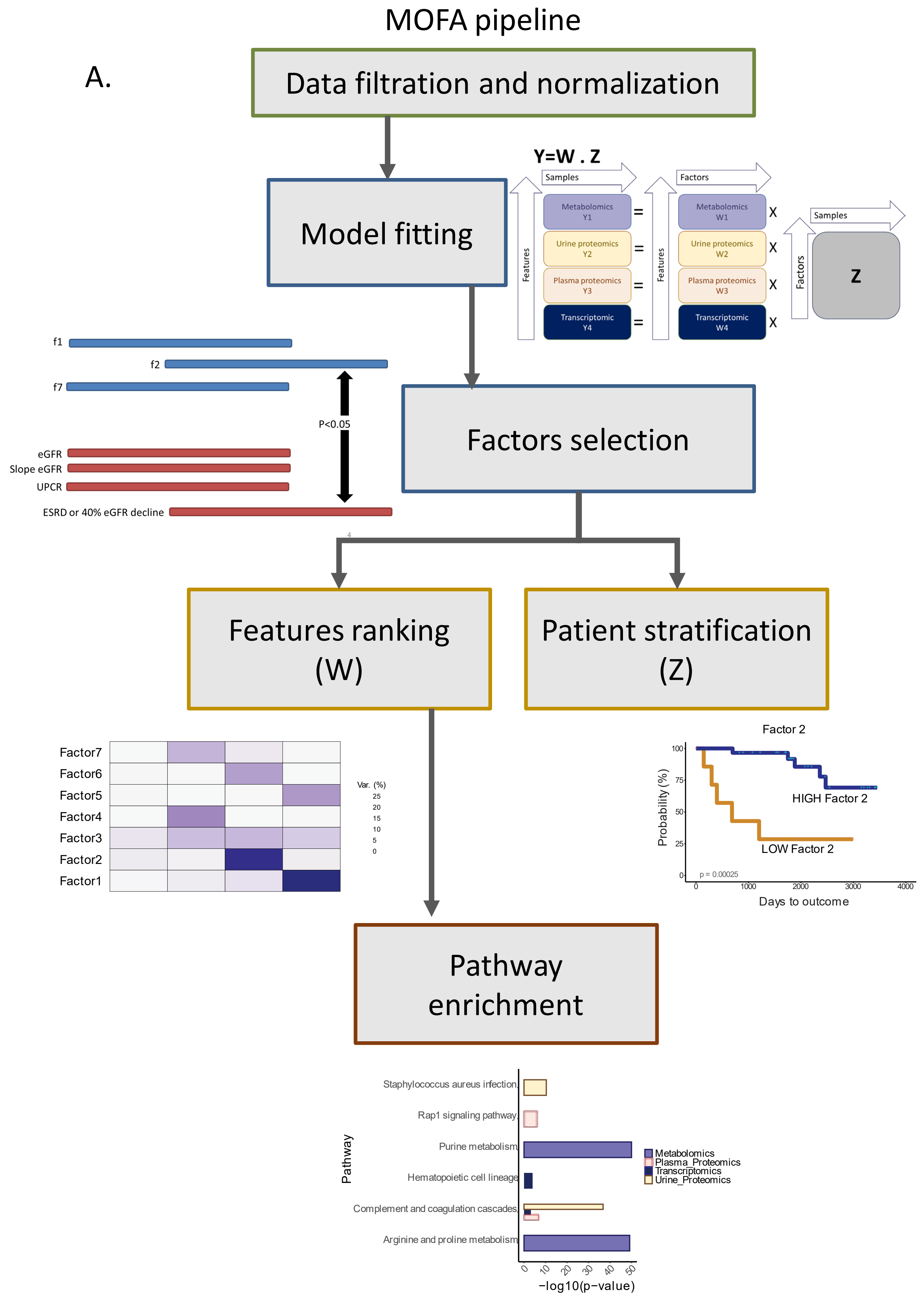
MOFA pipeline
A.
Data filtration and normalization
Model fitting
Y=W . Z
Samples
Features
Metabolomics Y1
Urine proteomics Y2
Plasma proteomics Y3
Transcriptomic Y4
=
Factors
Metabolomics W1
Urine proteomics W2
Plasma proteomics W3
Transcriptomic W4
X
Samples
Factors
Z
f1
f2
f7
P<0.05
eGFR
Slope eGFR
UPCR
ESRD or 40% eGFR decline
Factors selection
Features ranking (W)
Patient stratification (Z)
Factor7
Factor6
Factor5
Factor4
Factor3
Factor2
Factor1
Var. (%)
25
20
15
10
5
0
Factor 2
Probability (%)
100
75
50
25
0
HIGH Factor 2
LOW Factor 2
p = 0.00025
0
1000
2000
3000
4000
Days to outcome
Pathway enrichment
Staphylococcus aureus infection
Rap1 signaling pathway
Purine metabolism
Hematopoietic cell lineage
Complement and coagulation cascades
Arginine and proline metabolism
Pathway
Metabolomics
Plasma_Proteomics
Transcriptomics
Urine_Proteomics
0
10
20
30
40
50
−log10(p−value)

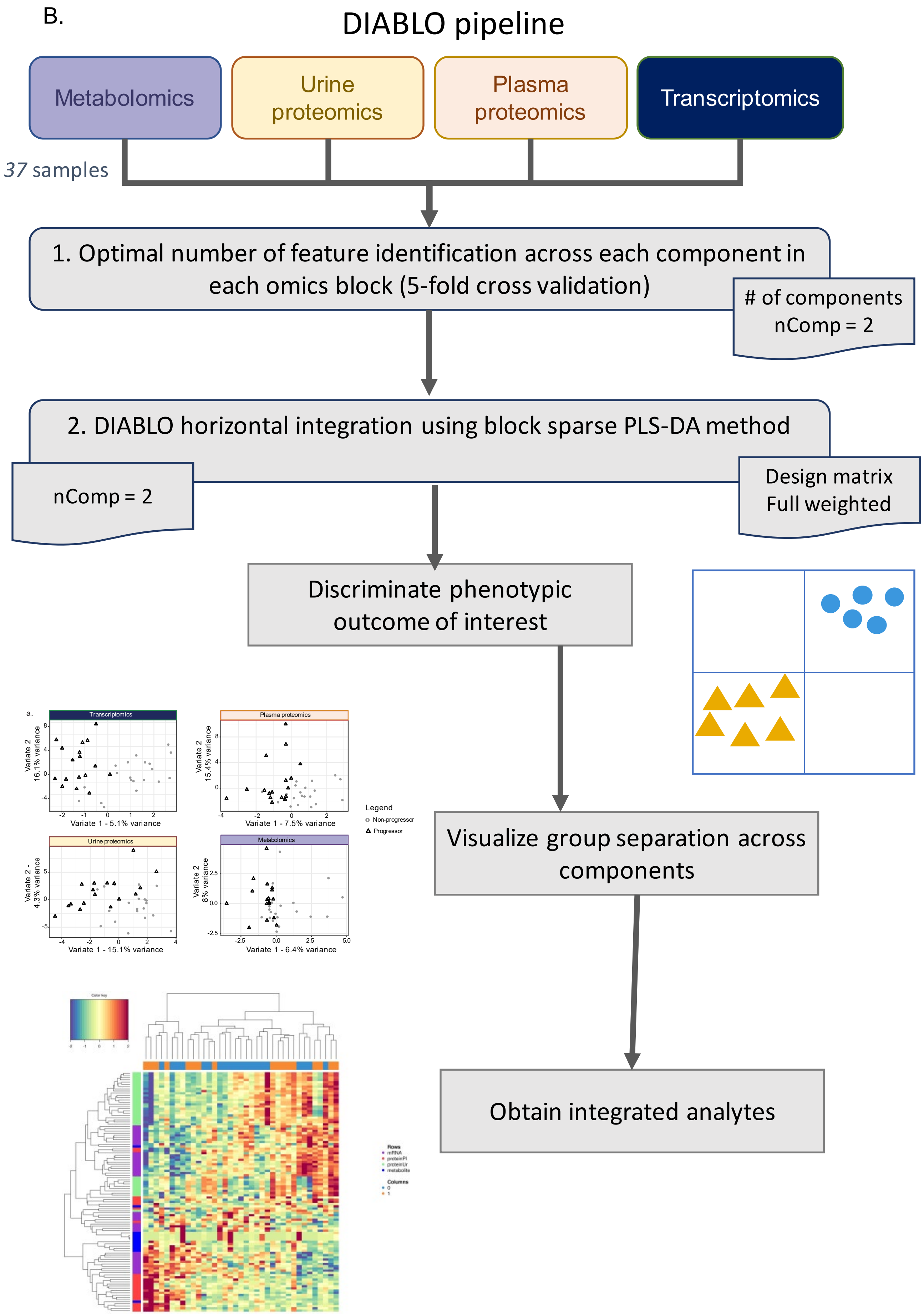

Supplementary Figure S2. Detailed pipeline of MOFA and DIABLO A. Pipeline for the MOFA algorithm. **B.** Pipeline for the DIABLO algorithm

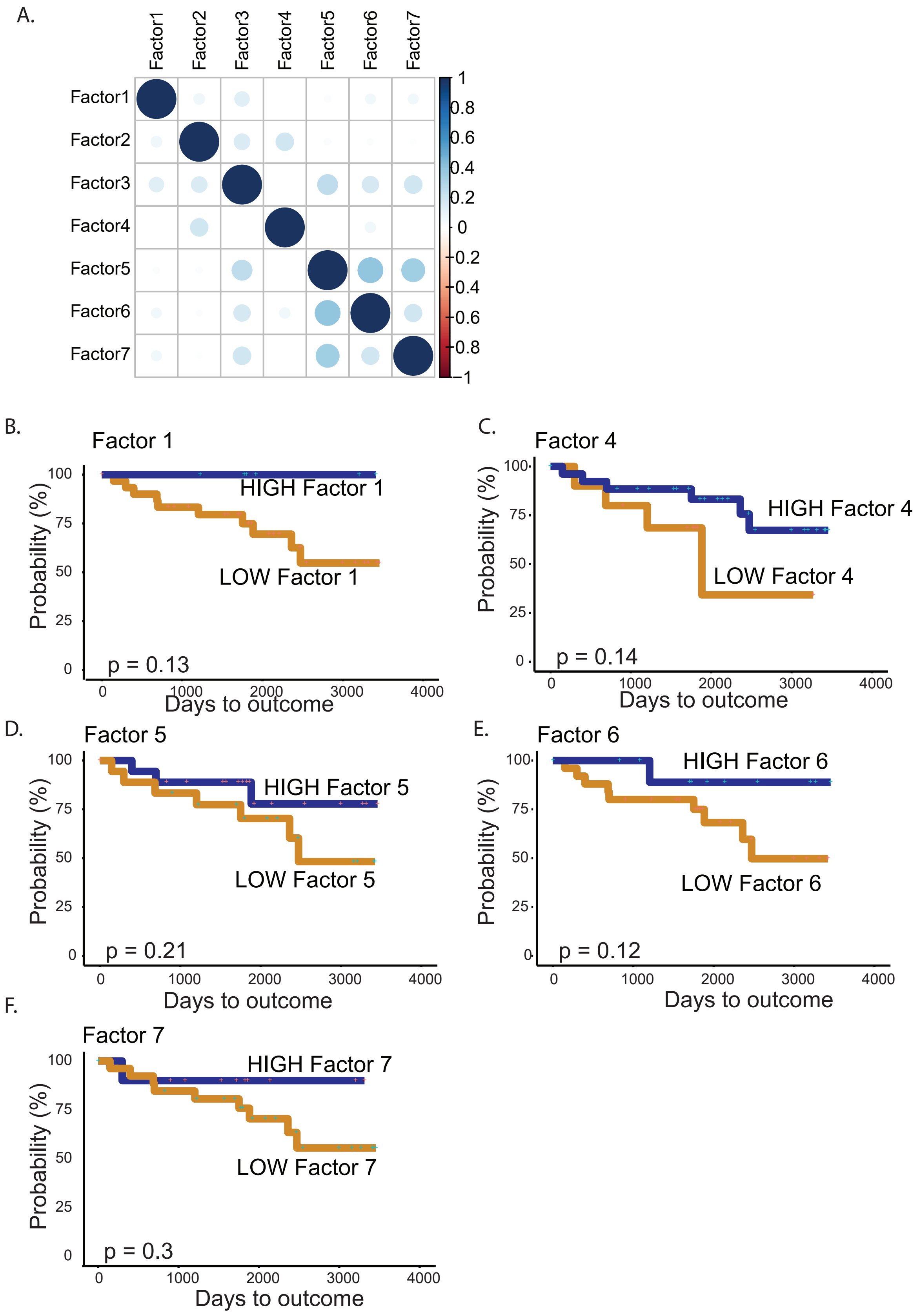

Supplementary Figure S3. **MOFA factors and its association with disease progression A.** Pearson correlation between MOFA factors. **B-F.** KM survival curve using the value of MOFA factors.

Supplementary Figure S4

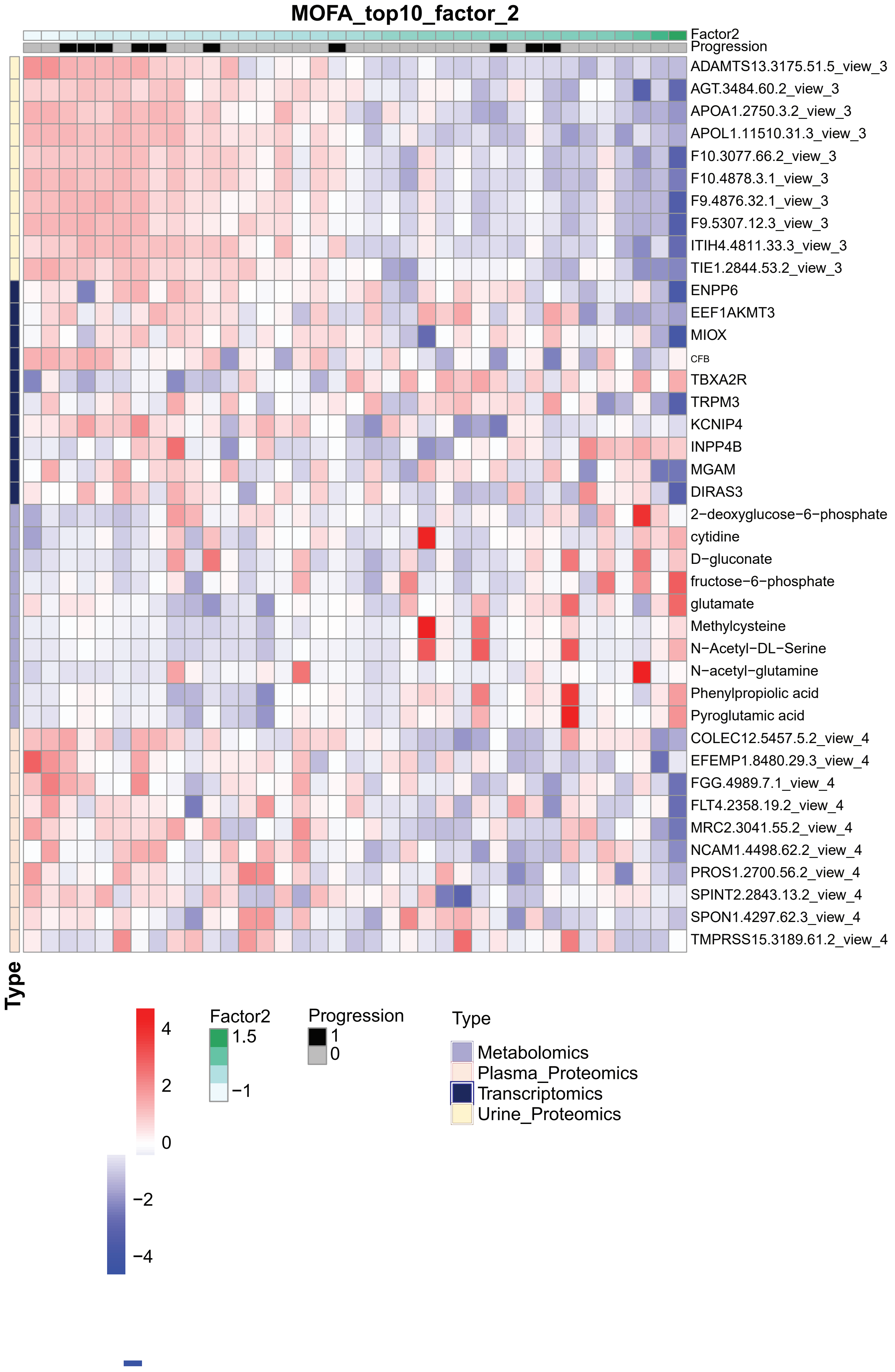

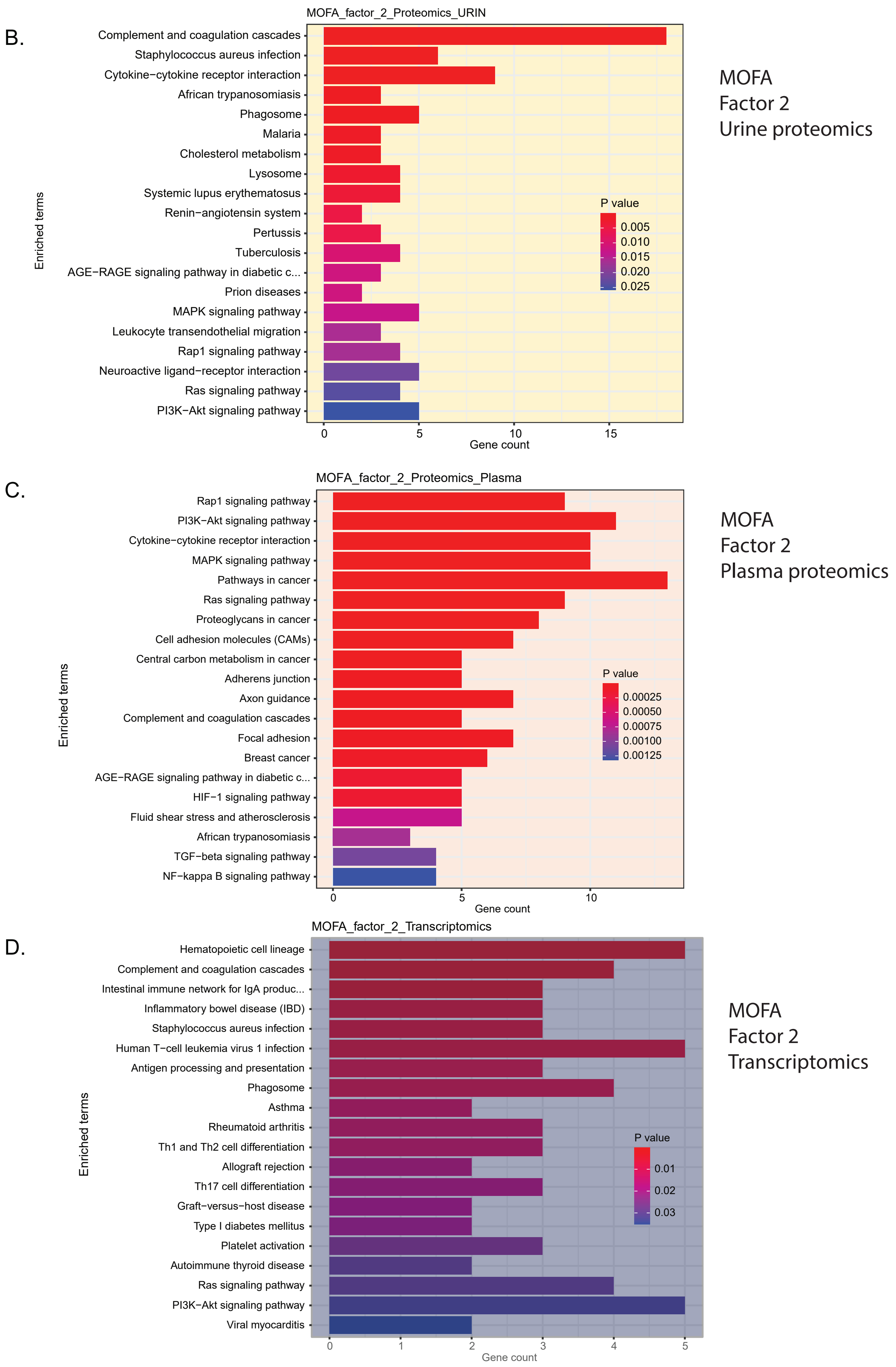

Supplementary Figure S4. Top features ranked by MOFA for factor 2 and corresponding enriched pathways A: Heatmaps depicting the top 10 ranked features contributing to the calculation of Factor 2 and Factor 3. C-E: Pathway enrichment analysis showcasing the top 100 features from each omics data type associated with Factor 2. F: Omics pathway enrichment specific to Factor 3. G-I: Pathway enrichment of the top 100 features from each omics data type linked to Factor 3. O: Heatmaps revealing the top 10 ranked features identified by DIABLO.

Supplementary Figure S5

A.

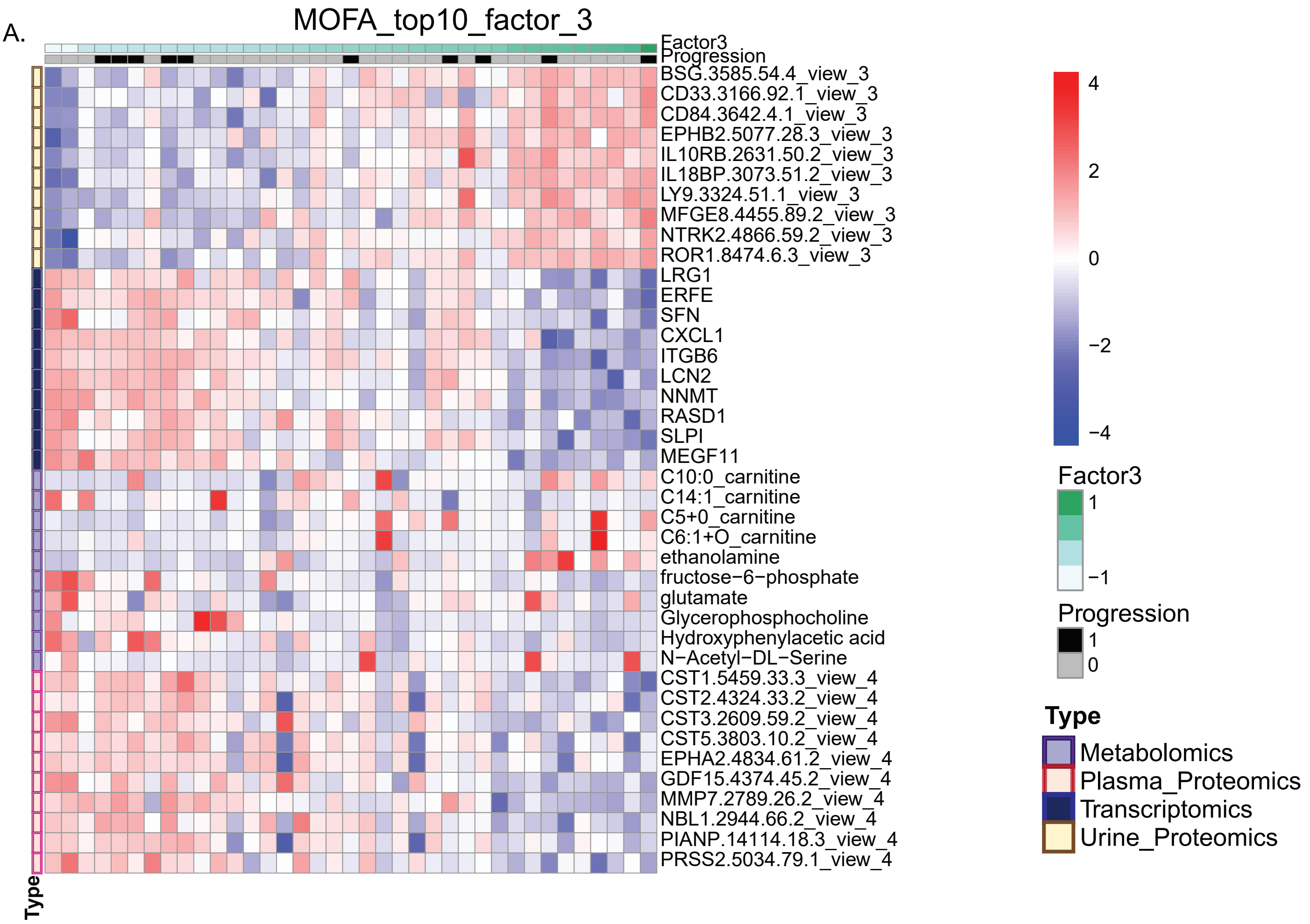

B.

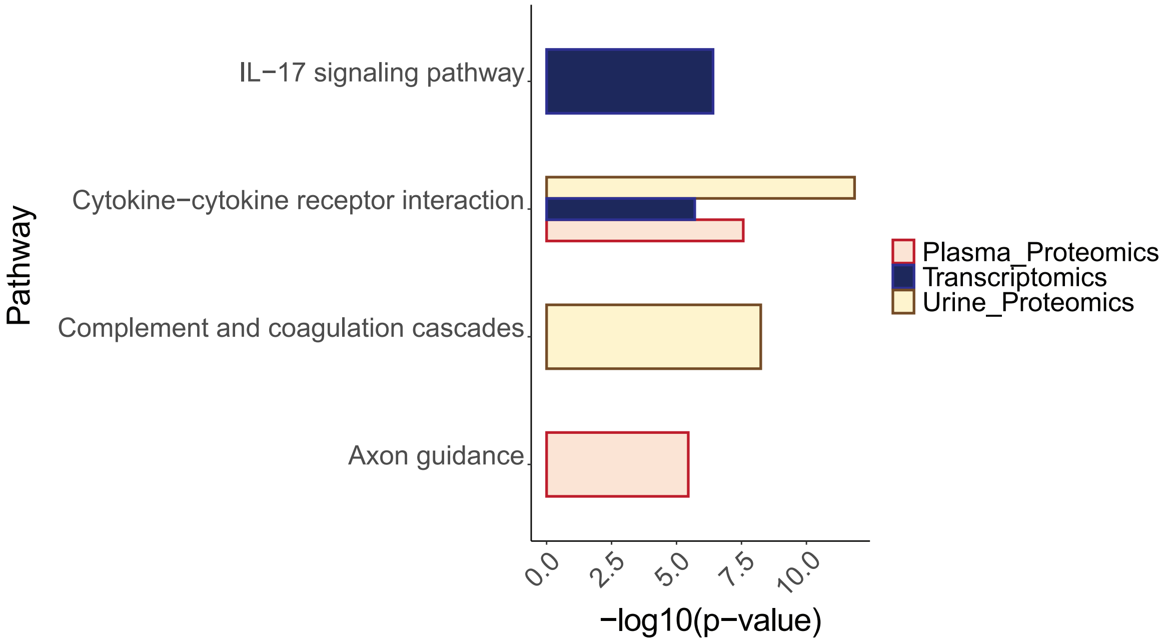

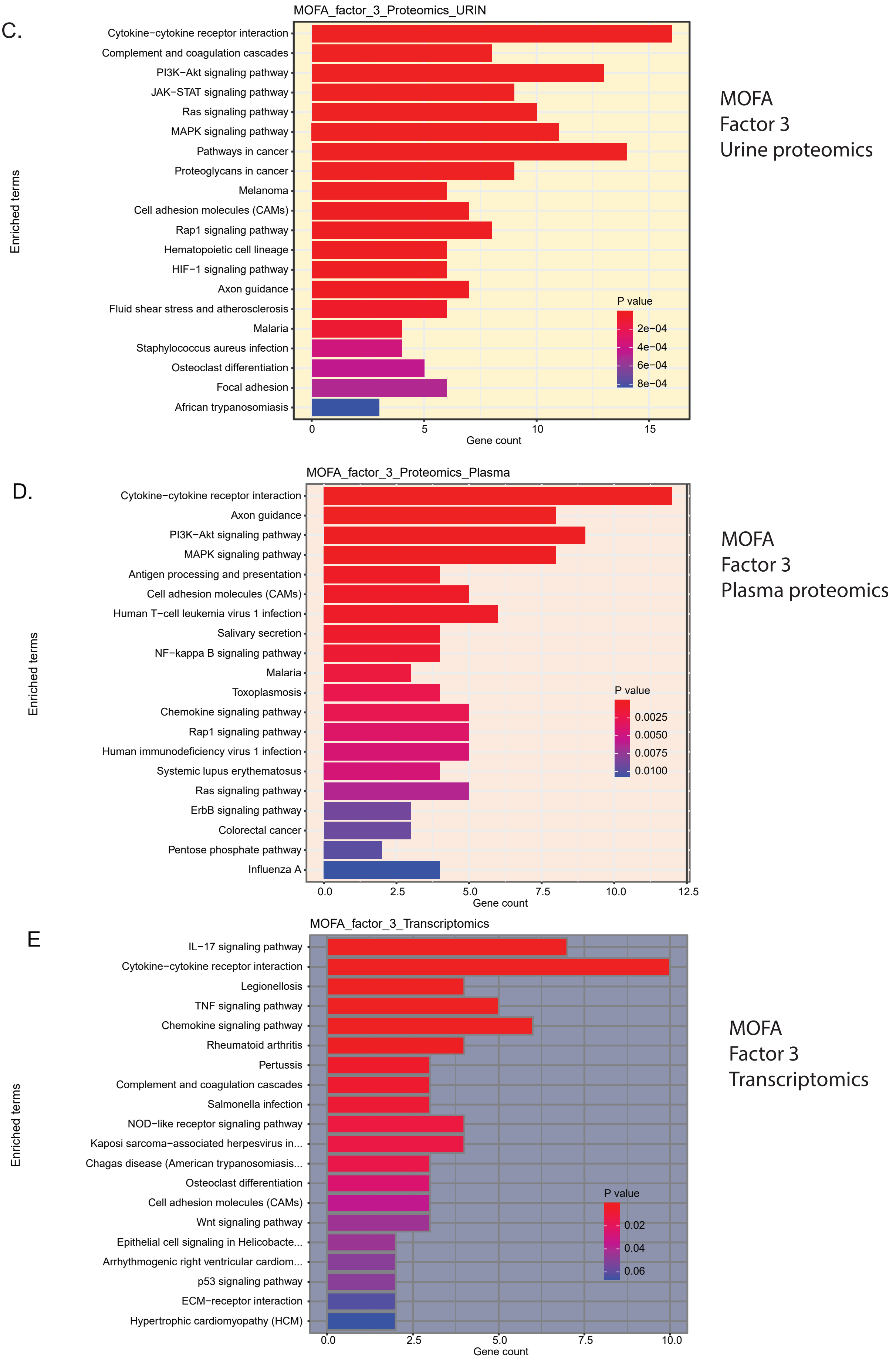

Supplementary Figure S5. Top features ranked by MOFA for factor 3 and corresponding enriched pathways. A: Heatmaps depicting the top 10 ranked features contributing to the calculation of Factor 2 and Factor 3. C-E: Pathway enrichment analysis showcasing the top 100 features from each omics data type associated with Factor 2. F: Omics pathway enrichment specific to Factor 3. G-I: Pathway enrichment of the top 100 features from each omics data type linked to Factor 3.

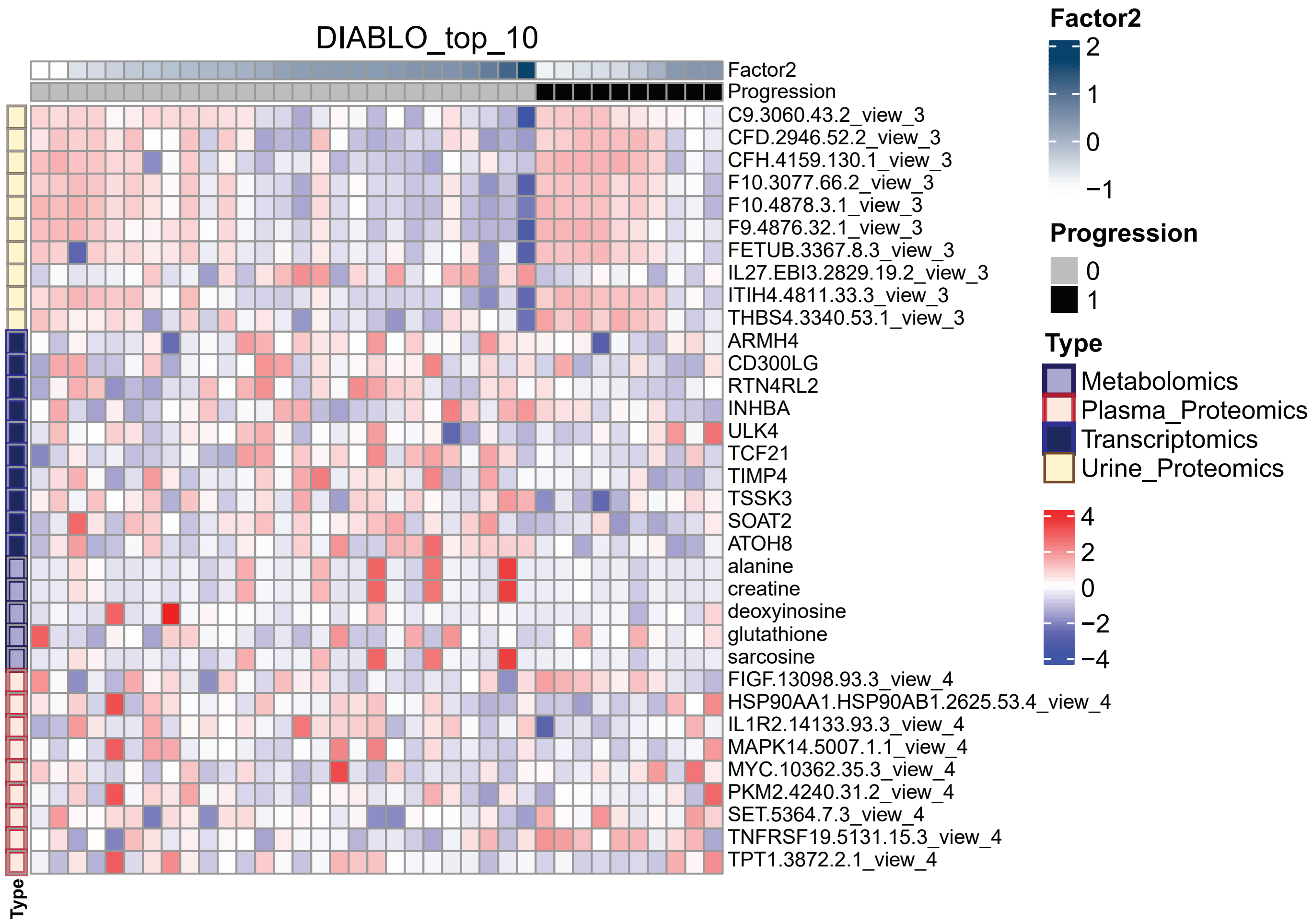

Supplementary Figure S6 Top features selected by DAIBLO for factor 2. Heatmaps of the top 10 ranked features identified by DIABLO.

Supplementary Figure S7

A. Up-regulated genes in CKD vs LD in ERCB cohort (Tub)

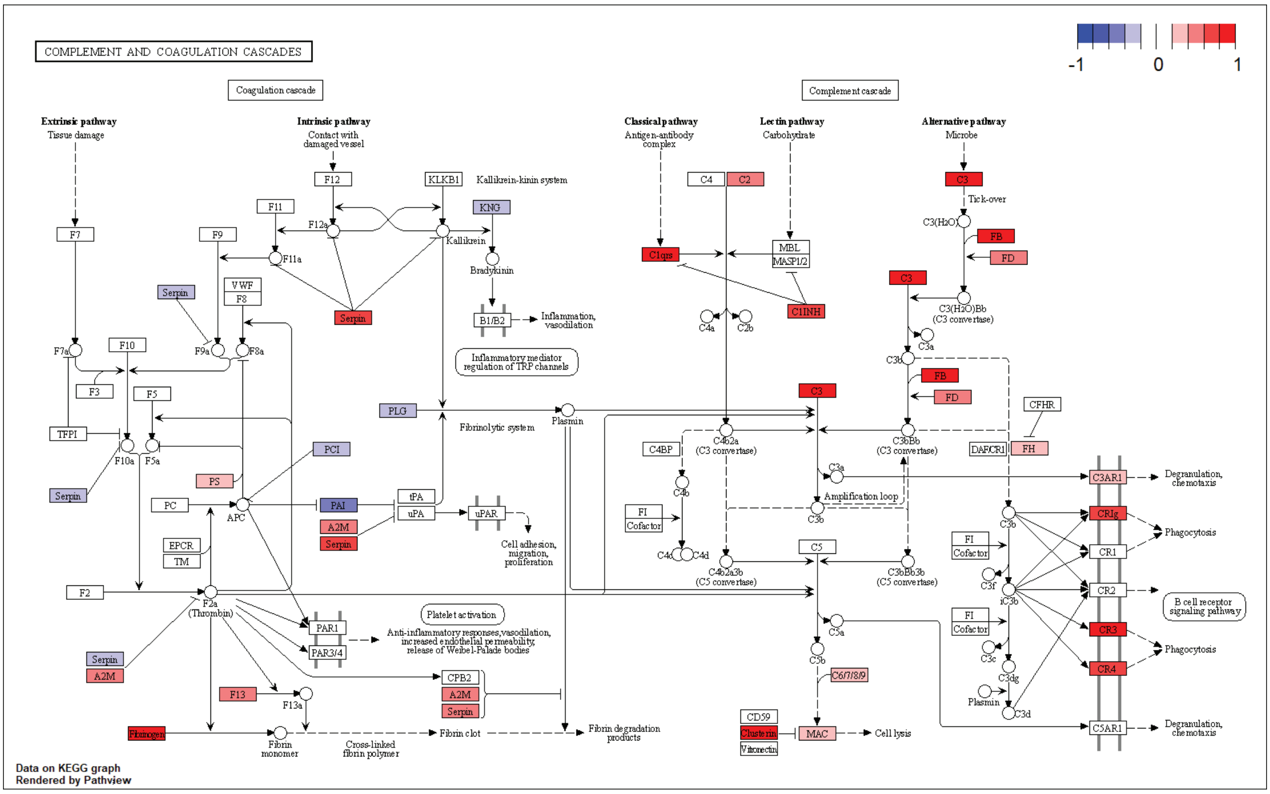

B. Up-regulated genes in CKD vs LD in ERCB cohort (GLOM)

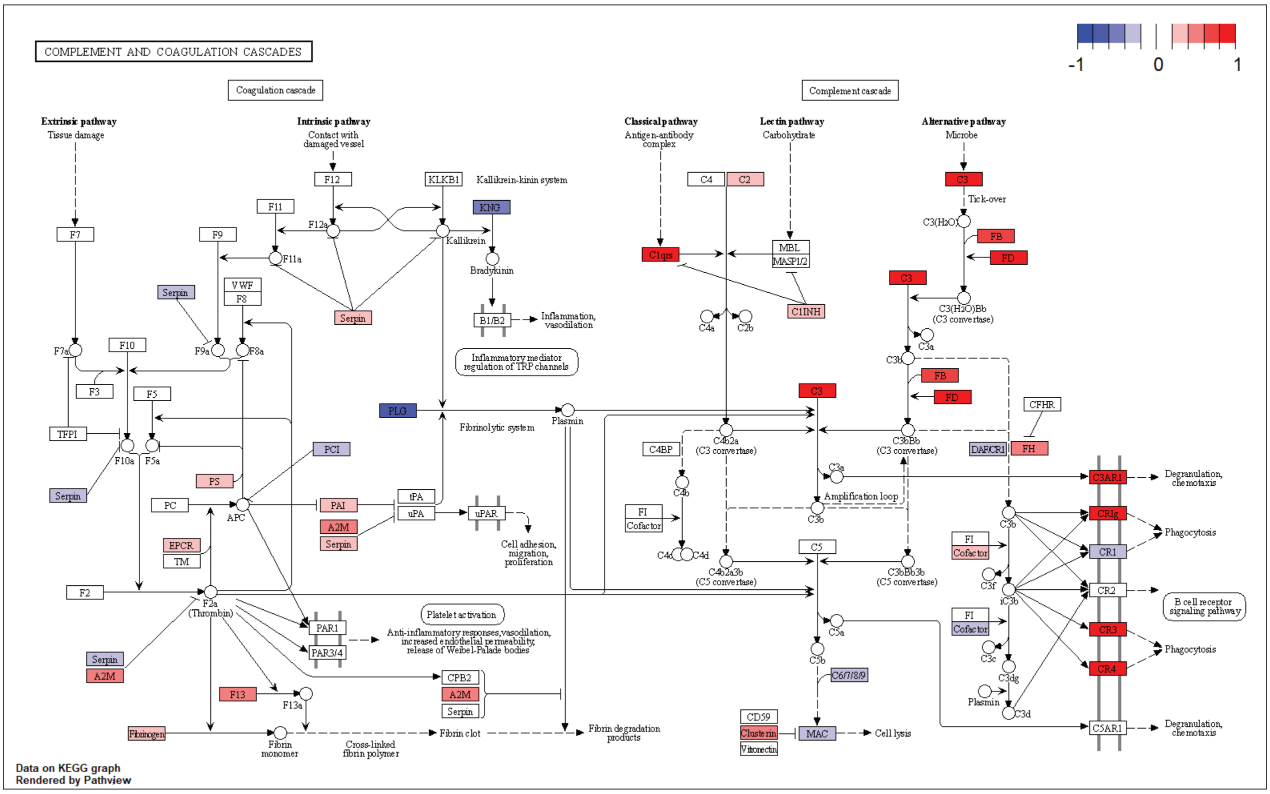

C. Correlated genes with eGFR in ERCB cohort (Tub)

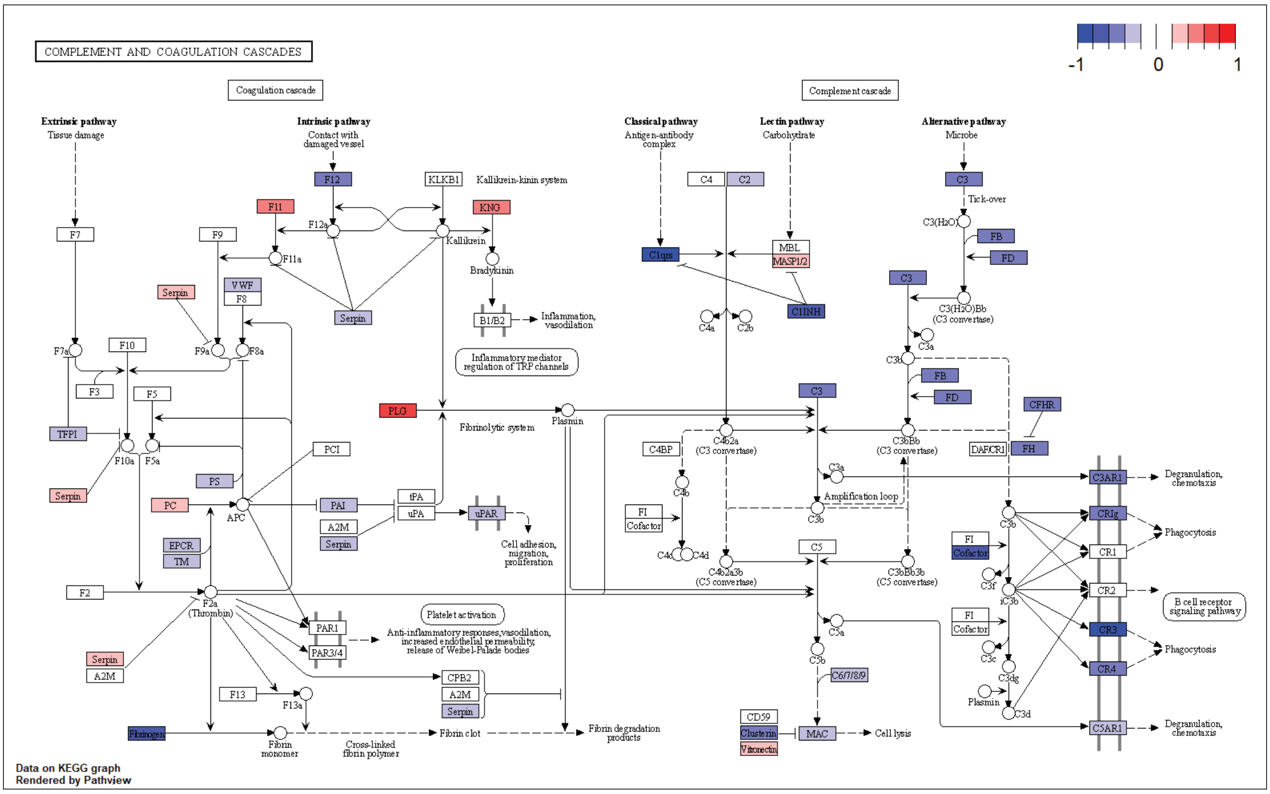

D. Correlated genes with eGFR in ERCB cohort (Glom)

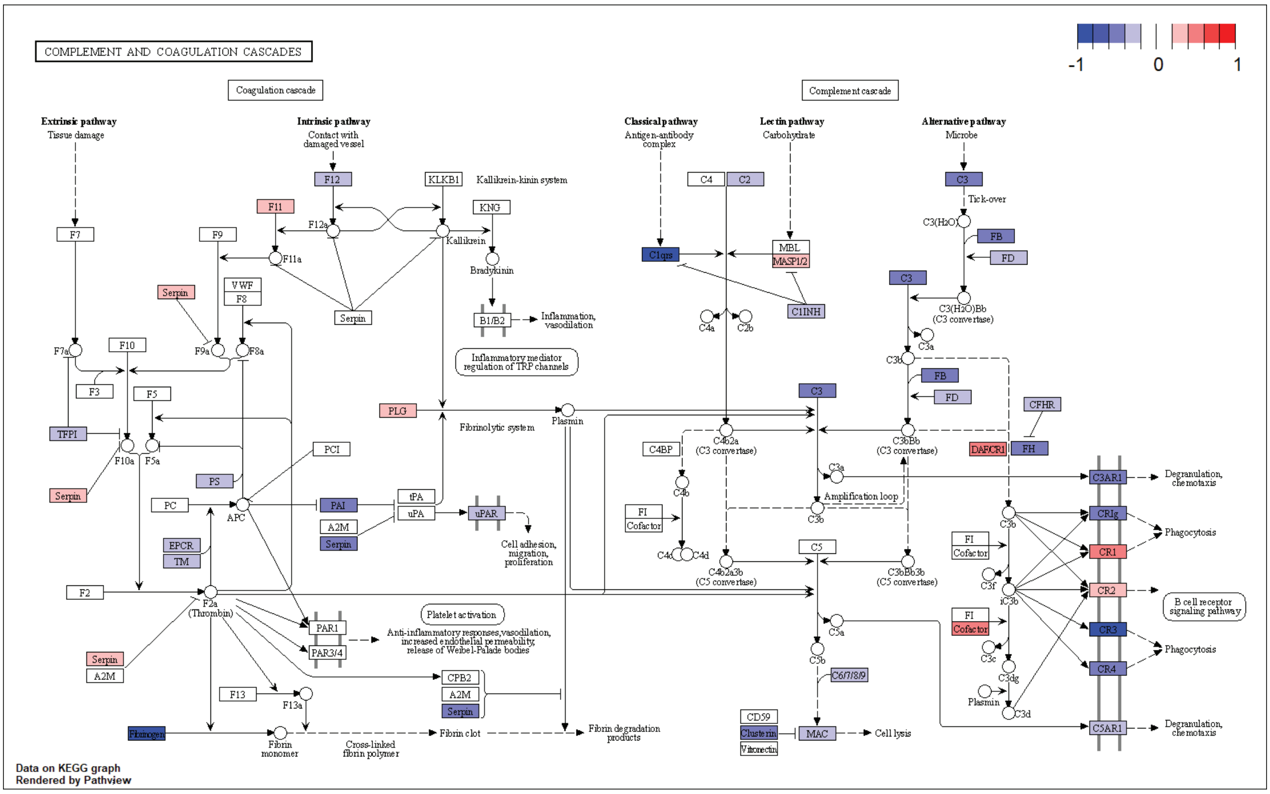

Supplementary Figure S7: Pathway of complement and coagulation cascade KEGG pathway. A. Color represents the logFC of significant (P adj <0.05) differentiated transcripts when comparing CKD with LD patients using transcriptomics data from TI and GLOM compartments **B.** in ERCB cohort. **C.** colour indicated the significant (P adj<0.05) Pearson correlation coefficient between complement transcripts and basal eGFR in the TI and **D.** GLOM compartment in ERCB cohort.
